# An Evidence-Based Serious Game App for Public Education on Antibiotic Use and Resistance: Randomized Controlled Trial

**DOI:** 10.2196/59848

**Published:** 2024-09-05

**Authors:** Zhilian Huang, Jing Teng Ow, Wern Ee Tang, Angela Chow

**Affiliations:** 1 Department of Preventive and Population Medicine Tan Tock Seng Hospital Singapore Singapore; 2 Clinical Research Unit National Healthcare Group Polyclinics Singapore Singapore; 3 Lee Kong Chian School of Medicine Nanyang Technological University Singapore Singapore; 4 Saw Swee Hock School of Public Health National University of Singapore Singapore Singapore

**Keywords:** serious game application, randomized controlled trial, antimicrobial resistance, antibiotic use, public education, mobile phone

## Abstract

**Background:**

The misuse and overuse of antibiotics accelerate the development of antimicrobial resistance (AMR). Serious games, any form of games that serve a greater purpose other than entertainment, could augment public education above ongoing health promotion efforts. Hence, we developed an evidence-based educational serious game app—SteWARdS Antibiotic Defence—to educate players on good antibiotic use practices and AMR through a game quest comprising 3 minigames and interaction with the nonplayer characters.

**Objective:**

We aimed to evaluate the effectiveness of the SteWARdS Antibiotic Defence app in improving the knowledge of, attitude toward, and perceptions (KAP) of appropriate antibiotic use and AMR among the public in Singapore.

**Methods:**

We conducted a 2-arm parallel randomized controlled trial, recruiting visitors aged 18-65 years from 2 polyclinics in Singapore. Intervention group participants had to download the SteWARdS Antibiotic Defence app (available only in English and on the Android platform) on their smartphones and complete the quest in the app. Participants took half a day to 2 weeks to complete the quest. The control group received no intervention. Knowledge questions on antibiotic use and AMR (11 binary questions) were self-administered at baseline, immediately after the intervention, and 6-10 weeks post intervention, while attitudes and perception questions (14 three-point Likert-scale questions) were self-administered at baseline and 6-10 weeks post intervention. We also collected participants’ feedback on app usage.

**Results:**

Participants (n=348; intervention: n=142, control: n=206) had a mean age of 36.9 years. Intervention group participants showed a statistically significant improvement in mean knowledge score (effect size: 0.58 [95% CI 0.28-0.87]) compared with controls after accounting for age, educational level, and exposure to advertisements on antibiotics and AMR. Intervention participants also showed a statistically significant improvement in mean attitude-perception scores (effect size: 0.98 (95% CI 0.44-1.52)) after adjusting for marital status and race. A majority of participants agreed that the “SteWARdS Antibiotic Defence” app improved their awareness on antibiotic use (135/142, 95.1%) and AMR (136/142, 95.8%). About 73.9% (105/142) of the participants agreed that the app is easy to use, 70.4% (100/142) agreed that the app was enjoyable, and 85.2% (121/142) would recommend the app to others.

**Conclusions:**

Our educational serious game app improves participants’ KAP on appropriate antibiotic use and AMR. Public education apps should be engaging, educational, easy to use, and have an attractive user interface. Future research should assess the effectiveness of interventions in facilitating long-term knowledge retention and long-lasting behavioral change.

**Trial Registration:**

ClinicalTrials.gov NCT05445414; https://clinicaltrials.gov/ct2/show/NCT05445414

**International Registered Report Identifier (IRRID):**

RR2-10.2196/45833

## Introduction

The World Health Organization declared antimicrobial resistance (AMR) as one of the top 10 global public health threats in 2019 [1]. Antibiotic-resistant bacteria can cause human infections that are harder to treat, leading to higher medical costs, decreased work productivity, and increased mortality [2-4]. By 2050, AMR is projected to cause 10 million annual deaths and up to US $100.2 trillion in economic losses worldwide if nothing is done to slow its progression [5]. The widespread misuse and overuse of antibiotics, often driven by the public’s lack of knowledge regarding appropriate antibiotic use and AMR, contribute to AMR progression [6,7].

The need to educate the public on appropriate antibiotic use and AMR is apparent, as better knowledge of antibiotic use was found to be associated with favorable antibiotic attitudes, which lowered the odds of expecting and receiving an antibiotic prescription [8]. Despite yearly public outreach [9] and multiple national educational campaigns on antibiotics and AMR [10], misconceptions about antibiotics—such as believing that antibiotics treat viral infections—continue to persist among the Singaporean population [7,8]. Traditional efforts to educate the public on antibiotic use and AMR have shown ambiguous effectiveness [11,12]. Hence, the lackluster effectiveness of existing efforts to prevent antibiotic misuse and overuse provides an impetus to explore novel public education methods beyond traditional modalities (eg, posters and pamphlets) [13].

Serious games have emerged as a promising tool for health education and health promotion across various fields recently [14]. This learning modality encompasses online and offline tools that use gamification—the use of game-playing elements—to provide an enhanced learning experience. Studies have shown promise of serious games in improving the short-term health knowledge, attitudes, and beliefs of young people, but the studies were too heterogeneous to prove the efficacy of this learning modality [15,16]. There were fewer serious games studies with older adults, but a web-based serious game co-designed with members of the public to raise pancreatic cancer awareness found a statistically significant improvement of pancreatic cancer awareness [17].

Serious games have also been used to promote good antibiotic use behaviors [16,18]. Preliminary evidence of serious games interventions on antibiotic use and AMR has shown promise in improving the knowledge of appropriate antibiotic use [16,19]. However, these studies were primarily focused on children and students. Adults have greater access to antibiotics and are more likely to pass down antibiotic use behaviors to the younger generation [13]. Hence, it is essential to educate young adults on appropriate antibiotic use. Existing studies also limited participants to a controlled environment with restricted playing time and may not fully capture real-world behavior. Furthermore, many studies overlooked the importance of long-term knowledge retention. The paucity of high-quality randomized controlled trials (RCTs) in the real-world setting highlights a pertinent gap in the evidence base of serious games for public education.

Singapore offers an excellent environment for evaluating serious game applications (apps) given its high smartphone penetration rate (92%) [20] and emphasis on digital transformation [21]. Hence, we developed an evidence-based serious game app—“SteWARdS Antibiotic Defence”—to deliver adult education on appropriate antibiotic use and AMR and aimed to evaluate its effectiveness in improving knowledge of, attitude toward, and perceptions (KAP) of antibiotic use and AMR in Singapore. We also sought user feedback on the “SteWARdS Antibiotic Defence” app.

## Methods

### Study Design

We conducted a parallel 2-arm RCT with a 6- to 10-week follow-up period. Participants in the intervention group had to download and complete the quest in the “SteWARdS Antibiotic Defence” app on their smartphones, while controls received no intervention.

### Study Setting

We recruited patients and their caregivers from 2 government-funded primary care clinics (ie, polyclinics) in Singapore. Recruitment occurred from January to March 2023, and follow-up was completed in early June 2023. The 23 polyclinics in Singapore manage 20% of Singapore’s primary health care needs by providing subsidized services such as medical treatment for acute and chronic conditions, vaccinations, and health education [22]. Participants seen at the polyclinics mainly have acute conditions that are managed outside the health care setting. Hence, while our study recruitment and baseline data collection occurred in the policlinics, the study intervention and follow-ups were administered outside the health care setting.

### Eligibility Criteria

Participants were 18-65 years of age, had access to an Android smartphone, and were English literate. We excluded those with visual or cognitive impairment, not proficient with smartphone apps, and those unable to install Android mobile apps from the study.

### Intervention

#### App Development

The “SteWARdS Antibiotic Defence” app is an evidence-based serious game mobile app developed by the study team, in collaboration with Temasek Polytechnic (a public tertiary institution in Singapore), to educate the public on appropriate antibiotic use and AMR. Players learn about appropriate antibiotic use and effective methods to recover from uncomplicated upper respiratory tract infections by interacting with the nonplayer characters (NPCs) releasing bite-sized information and playing the minigames in the app. We derived the educational content in the app from a rigorous review of knowledge gaps [7,8,23], antibiotic guidance [24], and inputs from health care professionals. The in-app messages were also adapted to the local context to ensure their relevance to players [25]. A few knowledge questions in the postgame and postintervention surveys were reverse scored to reduce to learning effect on knowledge gains. The bite-sized messages and knowledge questions can be found in Tables S1 and S2 in Multimedia Appendix 1.

**Figure 1:**
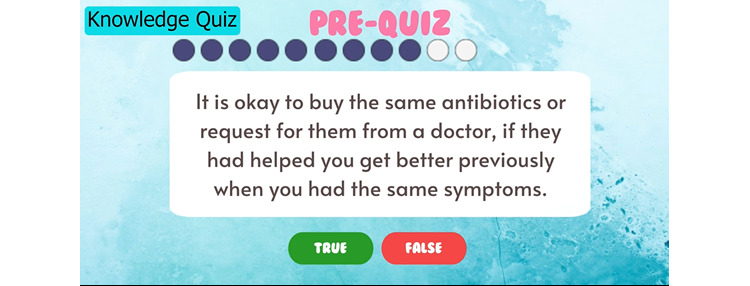
An example of a knowledge question embedded in the app. Participants are expected to complete 11 preintervention knowledge questions, followed by the game quest and 11 post–intervention knowledge questions to complete the intervention.

The pre- and postgame knowledge questions were embedded at the beginning and the end of the quest to reinforce knowledge retention (Figure 1). Completing the 11 baseline knowledge questions is a prerequisite to proceed to the minigames while completing the postgame knowledge questions is a prerequisite to fulfill the requirements of the intervention. Players are allowed to play the minigames repeatedly once they pass the level, but they can complete the pre-post survey only once. There is an in-app WhatsApp helpline for participants to contact the study team should they encounter technical difficulties. The feedback form link is also embedded in the app and appears after the completion of the postknowledge survey. Study team members (data collectors) provided intervention group participants with a onetime unique code to access the app during recruitment. The unique code allowed us to track the pre-post knowledge survey responses, time spent on the app, and interactions with the NPCs anonymously. We did not analyze the time spent on the app due to glitches that affected data quality.

#### Game Components

**Figure 2:**
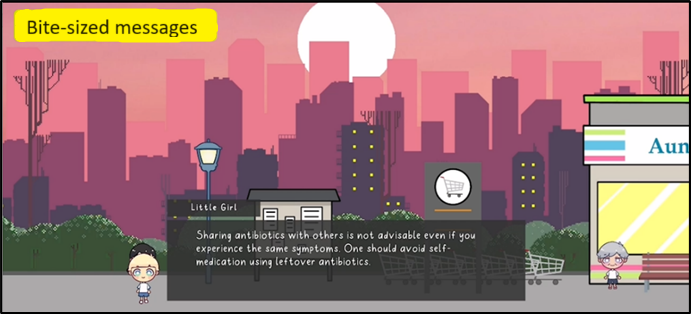
Interaction with a nonplayer character (NPC) at the park. Bite-sized messages on good antibiotic use practices were released through interactions with the NPCs. The rightmost NPC leads to the Tower Defence game.

**Figure 3:**
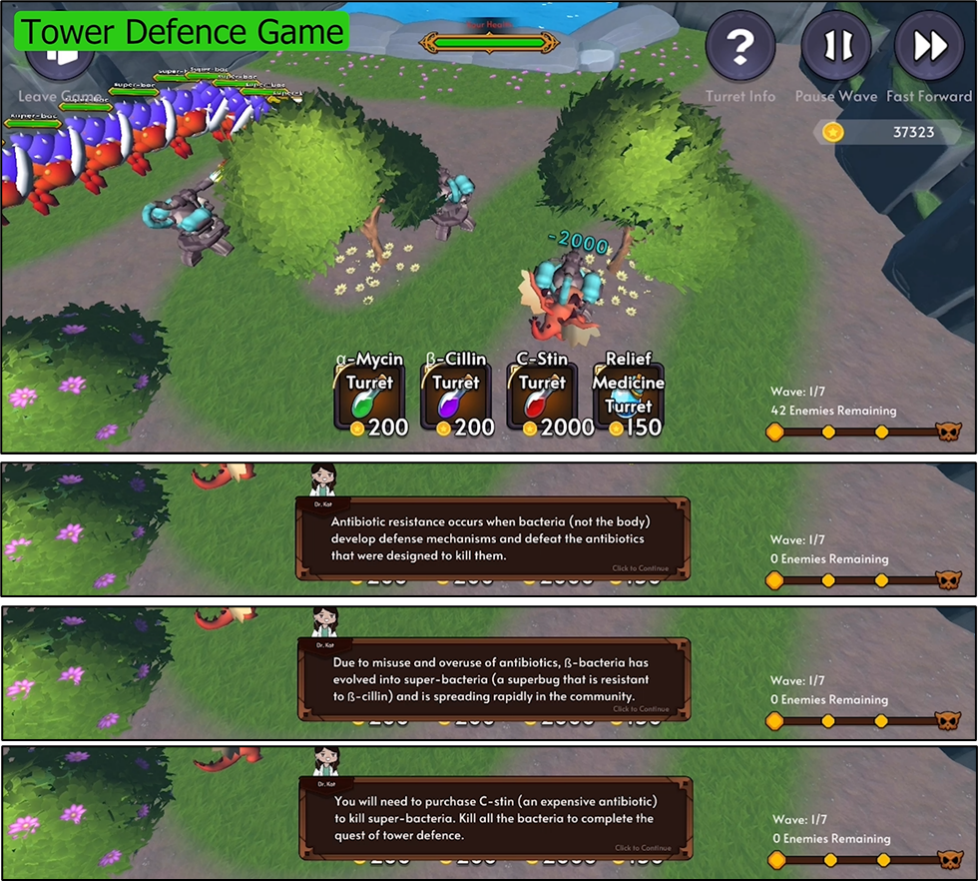
The Tower Defence game consists of 9 levels. The top figure shows the game interface with C-Stin turrets and the super bug (level 9). The subsequent figures show the messages released at the beginning of level 9 to teach players about the concept of antimicrobial resistance. Players have to choose the appropriate turrets and strategically place them at appropriate locations to effectively target their enemies (bacteria or viruses).

**Figure 4:**
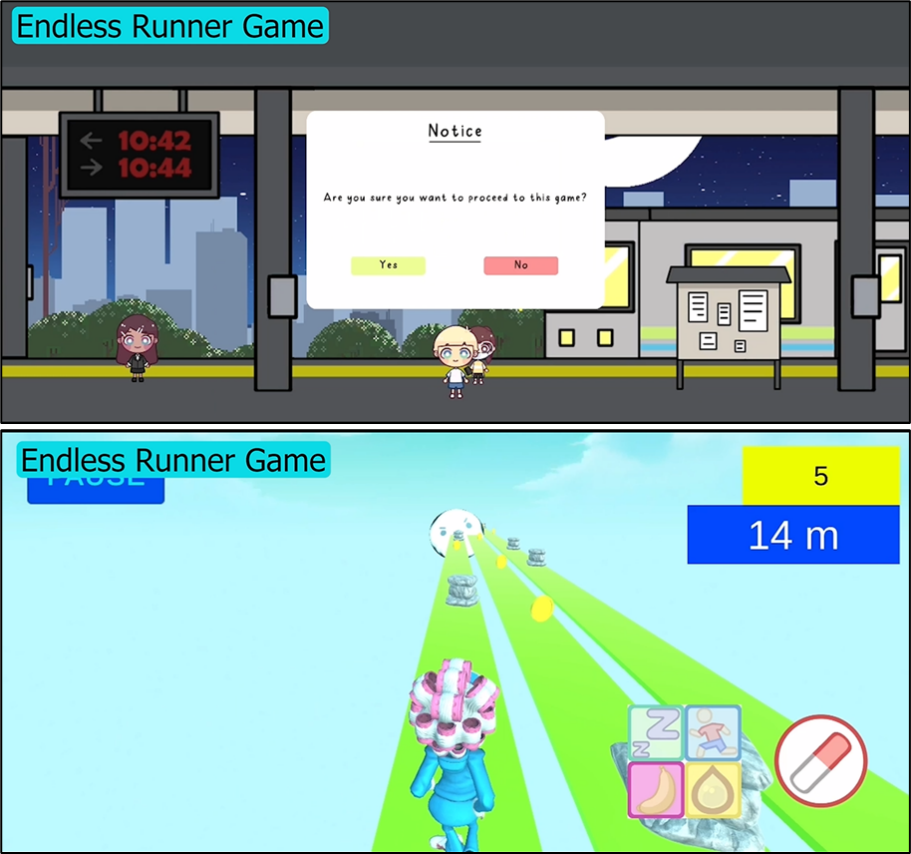
The Endless Runner game can be accessed via the train station (picture on top). The game reinforces the message that antibiotics are effective only against bacteria and not viruses. Players collect coins while running and use the antibiotic gun or immunity gun to shoot at bacteria or virus obstacles, respectively. Coins collected from the Endless Runner game can be used to purchase turrets in the Tower Defence game.

**Figure 5:**
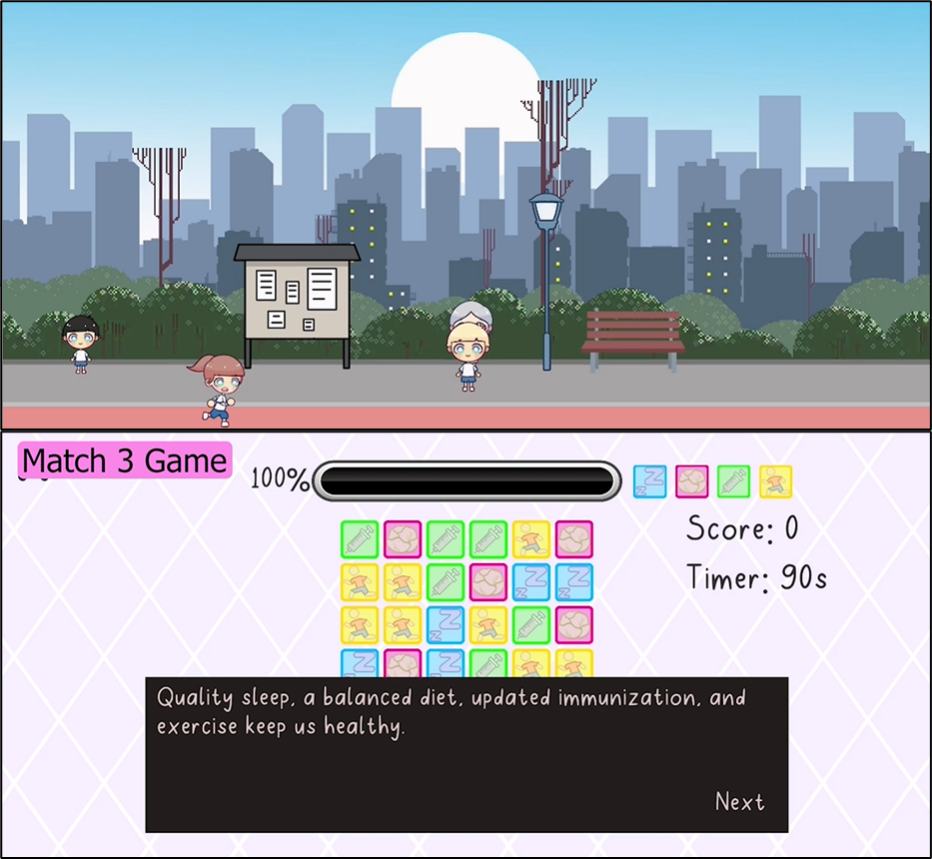
The Match3 game can be accessed via the park (picture on top). The game educates players on good health habits by focusing on sleep hygiene, nutrition, immunization, and physical exercise. Players are required to match 3 or more similar tiles within a specific time limit to successfully clear each of the 10 levels. Bite-sized messages are released before the start of each game to remind players of maintaining a healthy lifestyle.

There are 3 “worlds”—the “Supermarket,” “Park,” and “Train station”—that lead to the minigames in the app. Each “world” comprises 2 NPCs and a minigame. The supermarket is linked to the Tower Defence game; park is linked to the Match3 game; and train station is linked to the Endless Runner game (Figures 2-5).

There are 9 levels in the Tower Defence game to teach players about antibiotics and AMR. The first 2 levels teach players that antibiotics are effective against bacteria and the next 2 introduce the concept that antibiotics are bacteria-specific. Levels 5 and 6 educate players that antibiotics are not effective against viruses, levels 7 and 8 are reinforcements of previously taught concepts, and the last level educates players on antibiotic resistance by introducing a superbug that requires an expensive antibiotic (turret) to cure (kill the superbug). Players have to earn coins by passing Tower Defence levels and playing the Match3 and Endless Runner games.

The Endless Runner game is accessible via the train station. The game reinforces the message that antibiotics are effective only against bacteria and not viruses. Players collect coins while running to purchase turrets in the Tower Defence game, and use the antibiotic gun to shoot away bacteria obstacles and the immunity gun to shoot the virus obstacles.

The Match3 game is accessible via the park. The game educates players on good health habits by focusing on sleep hygiene, nutrition, immunization, and exercise. Players are required to match 3 or more similar tiles within a specific time limit to successfully clear each of the 10 levels.

Further details of the design and principles of the “SteWARdS Antibiotic Defence” app can be found in our published protocol [25]. Individual participants were followed up according to recruitment schedule from the point of recruitment. Participants took between half a day to 2 weeks to complete the intervention.

#### Outcomes

The primary outcome of interest was the change in KAP scores of antibiotic use and AMR. The secondary outcome was the level of user satisfaction for the “SteWARdS Antibiotic Defence” app. Primary outcomes were assessed for the intervention and control groups at baseline, immediately post intervention (intervention group only), and 6-10 weeks post intervention (from baseline for controls) via self-administered questionnaires. Knowledge was measured via 11 True/False questions, while attitudes and perceptions were measured via 14 questions on a 3-point Likert scale. Details of the KAP questionnaire are available in our published protocol [25]. For the intervention group, satisfaction with the “SteWARdS Antibiotic Defence” app was measured with 4 questions on a 5-point Likert scale and the reason(s) for recommending or not recommending the app to others, and suggestions for improvement were obtained via open-ended questions upon quest completion.

#### Sample Size

Assuming an effect size of 0.334 (95% CI 0.260-0.407) based on other serious games studies [18], we estimated a minimum sample size of 142 per group to detect a significant change in the knowledge score at a power of 80% and an a level of .05. We initially planned to recruit 200 participants per arm to account for a 30% attrition rate. We later increased the recruitment numbers to 240 per arm to account for a 40% attrition rate as we observed higher-than-expected dropout rates.

#### Study Procedures

Recruitment occurred on weekdays during the polyclinic’s opening hours. Staff members of the polyclinics were trained to recruit participants in their respective polyclinics. The data collectors would walk through every level of the polyclinic systematically to recruit participants daily. We also displayed digital recruitment posters around the polyclinics, so that interested visitors could contact the study team. However, only 2 participants contacted the study team via digital posters.

Data collectors approached the patient or the visitor nearest to them who seemed to fall in the eligible age category (18-65 years of age) to explain the study while systematically walking around the polyclinic. Next, the data collectors explained the study to the persons they approached and informed them of the possibility of being randomized into either the “survey group” (control) or the “app and survey” (intervention) group. Informed consent was obtained from interested participants before asking participants to pick a card to determine the group assignment. Participants knew which group they were assigned to as the intervention group participants were asked to download the app after their demographic data was collected. Baseline data collection occurred after randomization because the pre-post knowledge questions were built into the app. We collected participants’ KAP responses and demographic data at baseline and reimbursed them a small fee for completing the baseline surveys. All participants received the same amount of reimbursement at the baseline. Reimbursement was tiered to a higher value for the follow-up surveys to motivate participants toward task completion.

#### Baseline Data Collection

The baseline data collection process differed slightly between the intervention and control group participants. Intervention group participants had to complete the Attitudes-Perception demographic questionnaire on an iPad and install the “SteWARdS Antibiotic Defence” app on their smartphones. The data collector then provided the participant with a unique code to gain access to the app. Upon logging into the app, participants had to complete 11 baseline knowledge questions, followed by a walk-through of the basic features of the app. Controls completed the KAP and demographic questionnaire on an iPad.

#### Control Group Follow-Up

A study team member (ie, data collector) sends participants a detailed WhatsApp message 6 weeks after their recruitment date to inform them to complete the postintervention KAP questionnaire. The study team member sent a reminder if participants did not complete the questionnaire 2 weeks after the first WhatsApp message.

#### Intervention Group Follow-Up

Participants had to complete the “SteWARdS Antibiotic Defence” game quest and satisfaction survey and inform the study team via WhatsApp upon intervention completion. Six weeks after participants complete the intervention, a study team member sends a detailed WhatsApp message to inform them to complete the postintervention KAP questionnaire. A study team member reminded participants who did not complete the quest 2 weeks after recruitment and 2 weeks after sending the first KAP WhatsApp message.

#### Withdrawals and Loss to Follow-Up

Participants who became uncontactable during the study period were classified as loss to follow-up cases while those who indicated that they wished to be withdrawn from the study were considered as withdrawn cases. Withdrawn cases were asked to uninstall the app and were not further contacted.

#### Randomization

Eligible participants were 1:1 block randomized into the intervention or control group. Each block comprised 2 intervention and 2 control allocations. Participants determined their allocation by picking cards in the block, which was reset after all cards were drawn.

#### Blinding

Participants were not blinded to the intervention as the data collectors had to explain the "SteWARdS Antibiotic Defence" app to participants assigned to the intervention group. Apart from the data collectors, all other study team members (including the data analyst) were blinded to the group assignment.

### Data Analyses

We initially planned to take the intention-to-treat approach in analyzing the study data but eventually took the per-protocol approach, as our app usage data showed that participants who did not complete the game quest had too few interactions with the app to derive any benefit from the intervention. We standardized the total Knowledge and Attitude-Perception scores to 100% to illustrate the pre-post changes in scores. Correct answers are coded as 1 while wrong answers are coded as 0. Similarly, the “correct” attitudes are coded as 1 while neutral and “incorrect” attitudes are coded as 0.

Chi-square tests and Mann-Whitney *U* tests were used for univariate baseline comparisons. Weighted least squares regressions (weighted on preintervention scores) were used to assess the effect of the intervention on knowledge and KAP scores. The best regression model was chosen based on the lowest Akaike Information Criterion. All statistical assumptions were checked to ensure the accuracy of analyses. STATA (version 15; StataCorp LLC) was used for all statistical analyses.

Participant feedback was analyzed qualitatively using thematic analysis. OJT telephoned participants to elaborate on any ambiguous feedback and categorized the feedback into themes. ZH reviewed and discussed the categorization with OJT until a consensus was reached.

### Ethical Considerations

This study was approved by the National Healthcare Group Domain Specific Review Board (2022/00479) in Singapore. Informed consent was obtained from participants after the data collector had fully explained the study to them. Each participant was given a unique research ID linked to their personal details (eg, name and contact details). Only National Healthcare Group Domain Specific Review Board–approved study team members have access to participant data. All identifiable information is stored in password-protected files in the institutions’ server. Data collected from the surveys and app contained only the participants’ research IDs. Participants were reimbursed SGD 5 (US $3.82) for completing the baseline and demographic survey, SGD 10 (US $7.64) for completing the postintervention survey, and SGD 25 (US $19.10) for completing the app intervention and satisfaction survey (intervention group participants only).

## Results

### Participant Flow

**Figure 6:**
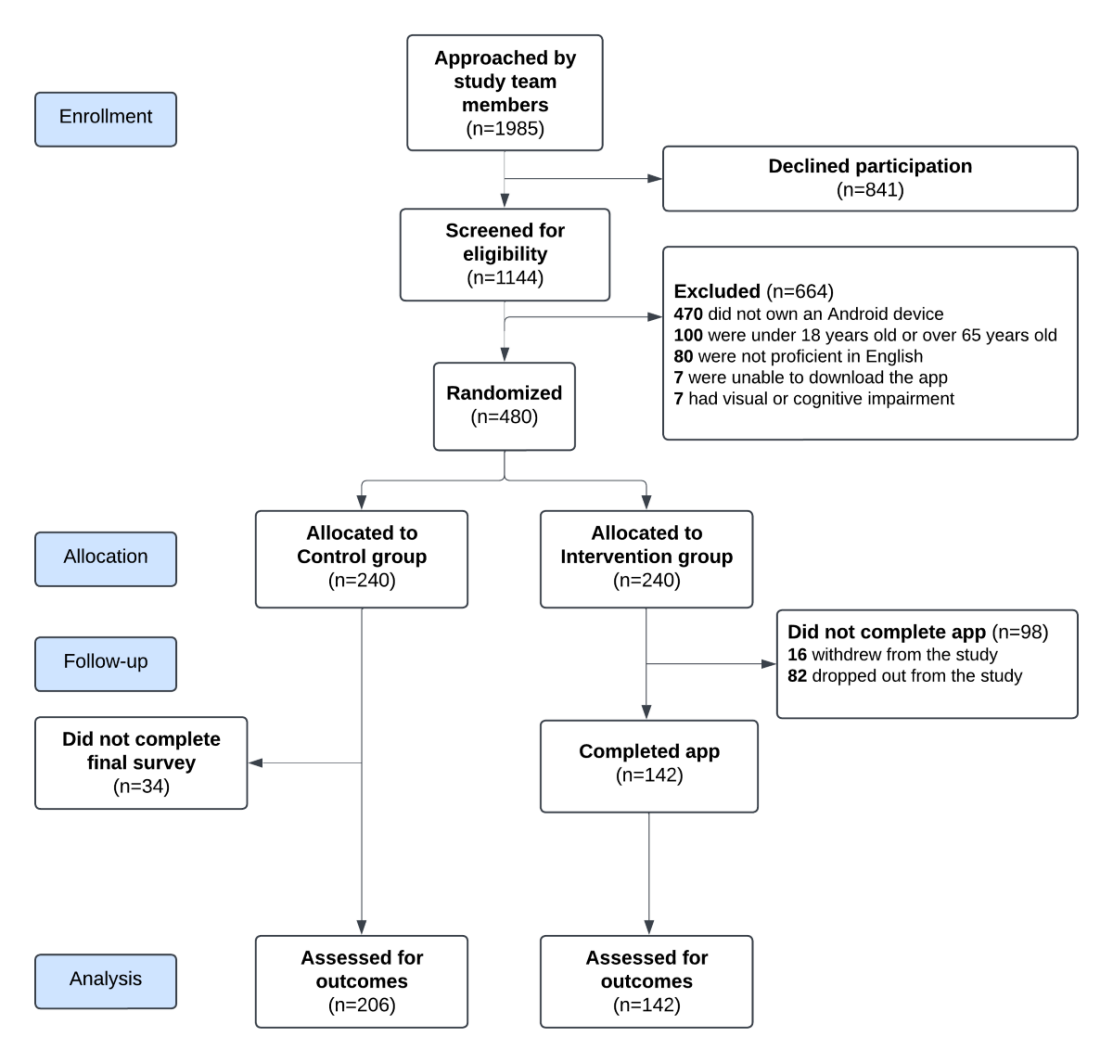
CONSORT (Consolidated Standards of Reporting Trials) flow diagram of a 2-arm randomized controlled trial conducted on participants recruited from government-funded primary care clinics between January 2023 and March 2023 in Singapore. A total of 1985 people were approached, 1144 were screened for eligibility, and 480 were recruited into the study and randomized equally into the control or intervention group. The outcomes of 142 intervention participants and 206 controls were eventually assessed.

We approached 1985 patients and visitors of polyclinics for 3 months, and 57.6% (1144/1985) of patients consented to take part in our study. Of these consenting participants, 41.1% (470/1144) of them did not own an Android phone, 8.7% (100/1144) did not meet our age requirements, 7.0% (80/1144) of them were not proficient in the English language, 0.6% (7/1144) of them had difficulty downloading the app, and 0.6% (7/1144) of them had visual or cognitive impairment. We block-randomized 42.0% (480/1144) of participants who were screened for eligibility into 2 study arms. Of these randomly assigned participants, 85.8% (206/240) of them in the control group and 59.1% (142/240) of them in the intervention group completed the study and had their outcomes assessed. About 40.8% (98/240) of intervention group participants did not complete the game quest (16 withdrew; 82 dropped out) in the app (CONSORT [Consolidated Standards of Reporting Trials] flowchart shown in Figure 6).

### Recruitment

Recruitment occurred between January 2023 and March 2023, and follow-up was completed by early June 2023. We ceased recruitment when we achieved 240 participants per arm.

### Baseline Data

Table 1 shows participants’ baseline characteristics. The randomization produced comparable groups as there were no significant between-group differences in participants’ sociodemographic characteristics at baseline. Participants had a mean age of 36.9 (SD 12.2) years. Our participant profile fits Singapore’s population profile in terms of ethnicity (252/348, 72.4% Chinese; 61/348, 17.5% Malay; and 24/348, 6.9% Indian), education level (128/348, 36.8% had tertiary education), and housing type (275/348, 79.0% residing in conventional public housing flats). There were also no significant between-group differences in the mean baseline knowledge score of 8.4/11 (SD 1.6), but the attitude-perception score was significantly higher in controls (mean 9.6, SD 2.6) than in intervention participants (mean 8.8, SD 3.0). In addition, 60.1% (209/348) of participants had no exposure to advertisements on antibiotic use at baseline.

The randomization produced comparable groups as there were no significant between-group differences in participants’ sociodemographic characteristics at baseline. Our participant profile fits Singapore’s population profile in terms of ethnicity and housing type.

**Table 1:** Baseline characteristics of the randomized controlled trial participants recruited from government-funded primary care clinics between January 2023 and March 2023 (N=348).

Baseline characteristics of respondents					All participants		Intervention group (n=142)		Control group (n=206)		*P* value
**Sociodemographic factors**											
	Age (years), mean (SD)			36.9 (12.2)		35.9 (12.0)		37.6 (12.3)		.21	
	**Sex, n (%)**										.64
			Male	181 (52.0)		76 (53.5)		105 (51.0)			
			Female	167 (48.0)		66 (46.5)		101 (49.0)			
	**Race, n (%)**										.79
			Chinese	252 (72.4)		106 (74.7)		146 (70.9)			
			Malay	61 (17.5)		23 (16.2)		38 (18.5)			
			Indian	24 (6.9)		8 (5.6)		16 (7.8)			
			Other races	11 (3.2)		5 (3.5)		6 (2.9)			
	**Educational level, n (%)**										.24
			Tertiary education^a^	128 (36.8)		47 (33.1)		81 (39.3)			
			Nontertiary education	220 (63.2)		95 (66.9)		125 (60.7)			
	**Marital status, n (%)**										.36
			Married	169 (48.7)		65 (45.2)		101 (49.3)			
			Unmarried	178 (51.3)		77 (54.2)		104 (50.7)			
	**Housing, n (%)**										.98
			HDB^b^ 3-room flat and below	81 (23.3)		33 (23.2)		48 (23.3)			
			HDB 4 or 5-room flat	194 (55.8)		80 (56.3)		114 (55.3)			
			Above HDB 5-room/private property	73 (21.0)		29 (20.4)		44 (21.4)			
**Knowledge, attitude, and perceptions on antibiotics and AMR^c^**											
	**Advertisement on antibiotic use, n (%)**										.34
			Had seen advertisements	139 (39.9)		61 (43.0)		78 (37.9)			
			Had NOT seen advertisements	209 (60.1)		81 (57.0)		128 (62.1)			
		Knowledge score, mean (SD) (maximum score: 11)		8.4 (1.6)		8.5 (1.6)		8.3 (1.7)		.16	
		Attitude-perception score, mean (SD) (maximum score: 14)		9.3 (2.8)		8.8 (3.0)		9.6 (2.6)		.004	

^a^Tertiary education: university and above.

^b^HDB flat: flats developed by Singapore’s public housing authority.

^c^AMR: antimicrobial resistance.

### Change in Knowledge Score

The mean standardized knowledge score improved from 77.6% at baseline to 82.4% postgame and 83.5% 6-10 weeks post intervention among intervention group participants, while controls had a modest improvement of 75.4% from baseline to 78.1% at 6-10 weeks post baseline (Figure S1 in Multimedia Appendix 1).

The multivariable regression analyses (Table 2) showed that intervention group participants had greater improvement in knowledge than controls (between-group differences: 0.58, 95% CI 0.28-0.87) after accounting for age, educational level, and exposure to advertisements on antibiotics and AMR.

**Table 2:** Multivariable weighted least squares regression analysis of the association between the intervention and knowledge score at 6-10 weeks postintervention.

Model variables			Outcome: 6-10 weeks of postintervention knowledge score					
			Effect size (95% CI)		*P* value		VIF^a^	
**Randomization group^b^ (reference: control)**								
	Intervention	0.58 (0.28 to 0.87)^c^		<.001		1.04		
**Knowledge**								
	Baseline knowledge score	0.24 (0.17 to 0.32)^c^		<.001		1.08		
**Age group (years; reference: 18-25 years)**								
	26-35	–0.75 (–1.19 to –0.31)^c^		.001		1.91		
	36-50	–0.43 (–0.90 to 0.03)		.07		2.49		
	51-65	–0.45 (–0.99 to 0.08)		.09		2.05		
**Marital status (reference: not married)**								
	Married	–0.32 (–0.67 to 0.03)		.07		1.51		
**Education status (reference: nontertiary education)**								
	Tertiary education	0.77 (0.45 to 1.09)^c^		<.001		1.13		
**Advertisement on antibiotics and AMR^d^ (reference: have not seen advertisements)**								
	Have seen advertisements	0.39 (0.09 to 0.69)^c^		.01		1.05		

^a^VIF: variance inflation factor.

^b^Intervention group participants had greater improvement in knowledge than controls after accounting for age, educational level, and exposure to advertisements on antibiotics and AMR.

^c^*P*<.05.

^d^AMR: antimicrobial resistance.

### Change in Attitude-Perception Score

The mean standardized attitudes-perception score improved from 62.6% at baseline to 77.7% post intervention among intervention group participants, while controls had a modest improvement of 68.9% from baseline to 71.9% post intervention (Figure S1 in Multimedia Appendix 1). The proportion of intervention group participants with the “correct” attitudes and perceptions increased for all 14 statements post intervention (Figure S2 in Multimedia Appendix 1). However, intervention group participants continue to have misconceptions about antibiotic use after completing the game quest. The proportion of participants with correct responses for the statement “I need antibiotics to recover from serious symptoms from the common cold and flu” increased from 29.6% (42/142) pre- to 46.5% (66/142) postintervention, and for “I need antibiotics if I continue to have flu symptoms after two weeks” increased from 22.5% (32/142) pre- to 38.7% (55/142) postintervention.

The multivariable regression analyses (Table 3) showed that intervention group participants had greater attitudes-perception improvement than controls (0.98 [0.44-1.52]) after accounting for marital status and ethnicity.

**Table 3:** Multivariable weighted least squares regression analysis of the association between the intervention and attitude-perception scores at 6-10 weeks postintervention.

Model variables			Outcome=6-10 weeks of postintervention attitude-perception score					
			Effect size (95% CI)		*P* value		VIF^a^	
**Randomization group^b^ (reference: control)**								
	Intervention	0.98 (0.44 to 1.52)^c^		<.001		1.30		
**Attitude-perception**								
	Baseline attitude-perception score	0.46 (0.39 to 0.53)^c^		<.001		1.26		
**Education status (reference: nontertiary education)**								
	Tertiary education	0.56 (–0.13 to 1.26)		.11		1.67		
**Ethnicity (reference: Chinese)**								
	Malay	–2.86 (–3.56 to –2.15)^c^		<.001		1.84		
	Indian	–1.28 (–2.19 to –0.36)^c^		.006		1.71		
	Others	–0.19 (–2.10 to 1.72)		.85		1.03		
**Marital status (reference: not married)**								
	Married	–1.39 (–1.99 to –0.78)^c^		<.001		1.67		

^a^VIF: variance inflation factor.

^b^Intervention group participants had better attitudes-perception improvement than controls after accounting for marital status and ethnicity.

^c^*P*<.05.

### App Satisfaction

The majority of participants agreed that the “SteWARdS Antibiotic Defence” app improved their awareness on antibiotic use (135/142, 95.1%) and AMR (136/142, 95.8%). About 73.9% (105/142) of the participants agreed that the app is easy to use, 70.4% (100/142) agreed that the app was enjoyable, and 85.2% (121/142) would recommend the app to others (Table 4).

**Table 4:** Satisfaction in using the “SteWARdS Antibiotic Defence” app (N=142).

App satisfaction			Responses, n (%)
**User experience^a^: How far would you agree that the app:**			
	Improved awareness on antibiotic use	135 (95.1)	
	Improved awareness on antimicrobial resistance	136 (95.8)	
	Is easy to use	105 (73.9)	
	Is enjoyable to use	100 (70.4)	
**User satisfaction**			
	Would recommend the app to others	121 (85.2)	

^a^User experience was measured on a 5-point Likert scale. We report the proportion of respondents who agreed with the statements on user experience. The majority of participants agreed that the “SteWARdS Antibiotic Defence” app improved their awareness on antibiotic use and AMR. More than 70% agreed that the app is easy to use, enjoyable, and would recommend the app to others.

Participants who would recommend “SteWARdS Antibiotic Defence” found the app user-friendly, fun, enjoyable, and interesting. Some participants also lauded the app’s unique learning modality and felt that the intervention addressed an important public health topic, raised their awareness of AMR, and improved their knowledge on appropriate antibiotic use (Table S3 in Multimedia Appendix 1).

It provides information about antibiotic resistance in an easy to understand, engaging way.

It is much more engaging compared to reading or [looking] through long videos.

Participants who would not recommend the app mainly had a poor user experience and were frustrated with technical difficulties in navigating the app. Some technologically astute players found the game boring, repetitive, and unfulfilling.

There were technical issues that made completing the game annoying.

One participant found no value in recommending the app to friends or family member working in health care. Those who found the games difficult to play (mostly older adults) or experienced usability issues (eg, small fonts) also expressed dissatisfaction with the intervention.

Seniors may not be able to understand how to play the games.

### Suggestions for App Improvement

Suggestions to improve the quality of “SteWARdS Antibiotic Defence” include the following (Table S4 in Multimedia Appendix 1):

#### Refining Game Mechanics

Participants pointed out that the minigames should focus on reinforcing concepts the study team intended to teach. For example, 1 minigame leans toward self-care instead of teaching about antibiotics use and AMR. Some participants also suggested improving the game’s instructional clarity, calibrating the in-game economy to optimize the game’s difficulty level, and increasing the number of interactive elements.

The upgrades in the Tower Defence Game is overpowered and makes it too easy.

Let users know how much the coins earned from “endless runner” are worth. This ties into the other game “tower defence” because you need coins to build the towers.

#### Enhancing the User Interface

Participants suggested having a consistent art style, larger word fonts to cater to older adults, and graphics with a closer resemblance to bacteria and viruses.

Maybe the educational message displayed before each game can be read out.

The 3D games can have better art style to match with the other 2D games and environment.

#### Having More App Features

Suggested app features include adding music and sound effects within the minigames, allowing players to create personal profiles, and in-app game demonstrations.

More sound effects would be nice.

#### Bug Fixes

Participants suggested to improve the app’s responsiveness, sensitivity, speed, and fixing the glitches.

Developer to look into the app responsiveness and the graphics.

## Discussion

### Main Findings

We evaluated the effectiveness of an evidence-based serious game mobile app in improving the KAP of appropriate antibiotic use and AMR among Singapore residents. To our knowledge, this is the first RCT assessing the effectiveness of a serious game mobile app intervention for public education on appropriate antibiotic use and AMR. We also assessed the effectiveness of the app in short-term knowledge retention at 6-10 weeks post intervention, which has often been overlooked. Furthermore, we assessed the app in a real-world context in the community and not in the controlled environment of an experimental setting.

We observed a statistically significant effect size of 0.58 (95% CI 0.28-0.87) in knowledge improvement, which concurs with other serious game studies on chronic disease management among young people [26] and healthy lifestyle promotion [27]. However, a similar Singapore study on dengue prevention observed significant improvements of participants’ knowledge, attitudes, and practices from baseline but no between-group mean differences [28]. The difference in observations is likely attributed to the exposure of relevant information sources among controls in the dengue study, which attenuated the effects of the app intervention. Tertiary-educated adults were positively associated with higher postintervention scores. They may fare better at learning the more difficult AMR concepts as studies have shown that AMR concepts are hard to teach. Participants with no or limited understanding of the etiology of antibiotic resistance would face difficulty connecting AMR with antibiotic misuse [29]. Furthermore, misconceptions about antibiotic use are generally hard to rectify.

We also observed a statistically significant effect size of 0.98 (95% CI 0.44-1.52) in attitude-perception improvement with the “SteWARdS Antibiotic Defence” app. Other studies have found mixed results in the effectiveness of serious games in improving the adoption of healthy behaviors, such as fruit and vegetables intake among adolescents [15]. The effectiveness of serious games in changing attitudes and perceptions are hard to measure as studies are often multifaceted. Therefore, it is essential to have well-designed interventions targeted at the correct population, well-accepted by participants, and with attainable goals for interventions to be successful. While we recruited participants between 18 and 65 years of age, the app was originally developed for young adults as this group of people was found to have the lowest knowledge in antibiotic use and AMR [7].

Our “SteWARdS Antibiotic Defence” app received an overall positive rating, but the ratings should be interpreted with caution as the satisfaction survey was only completed by participants who finished the game quest in the app. It is unclear whether participants dropped out of the intervention due to dissatisfaction with the app or a lack of time to complete the quest. Other studies assessing the effectiveness of serious games in health education have observed high satisfaction among participants [30,31]. However, there is limited evidence on the satisfaction with health education delivered via serious games in the general population and even fewer studies report participant satisfaction. Hence, the feedback and suggestions we gathered from participants are valuable in identifying population-specific shortcomings of the intervention and informing app enhancements that could increase the success of intervention scale-ups.

Despite a modest improvement in KAP scores in the intervention group, improved health literacy may not translate to desired behavioral change [13,32]. Health literacy interventions have led to improved health outcomes in some studies, but the links between health literacy improvement and behavioral change have not been established with higher-quality studies [33]. Effective interventions are also integral to attaining successful behavioral change. One way to improve the learning outcomes, suggested by our participants, is to weave the intended learning concepts into the game mechanics rather than displaying informational text boxes for players to read. Such enhancements would possibly improve user engagement and lead to better learning outcomes [34].

A few limitations exist in our study. First, measurement errors in KAP scores might occur if intervention group participants had requested their friends or family members to help with study completion. Nonetheless, controls could also have had help in completing the postintervention survey. Therefore, any bias which attenuates the intervention's true effect is likely toward the null. Second, there is a ceiling effect for participants with near-perfect baseline scores, which could attenuate the positive effects of the intervention. Third, we randomized participants prior to baseline data collection because the knowledge questions were embedded in the app. Fourth, there may be a learning effect in the intervention group as these participants completed the knowledge questionnaire 3 times. Hence, the positive effects observed in the intervention group could be a combination of the serious game and learning effect from the immediate postgame survey. Including a study group with a traditional form of learning (eg, brochures) may enable us to assess the effect of the serious game app more accurately. Finally, we included only attendees at 2 government-funded primary care clinics (polyclinics). Despite the limitations in recruitment, the sociodemographic distribution of our participants is representative of the Singaporean population. Hence, our findings are generalizable to the rest of the country.

In addition, we noticed several older adults rejecting study participation due to a lack of interest in completing the mobile app quest. Their lack of interest implies an additional effort to change their mindsets, and serious game interventions may not be the best modality for educating older adults on public health issues. Reminders were also integral in driving the app completion rate. Hence, the actual uptake rate may be lower in the population. Future research should assess the effectiveness of interventions that facilitate long-term knowledge retention and long-lasting behavioral change in antibiotic use and AMR [35].

### Conclusions

Our educational serious game app improves participants’ KAP on appropriate antibiotic use and AMR. Public education apps should be engaging, educational, easy to use, and have an attractive user interface. Interventions should also aim to achieve long-term knowledge retention and long-lasting behavioral change.

## Appendix

Multimedia Appendix 1 Additional materials to support the findings of the study.

Multimedia Appendix 2 CONSORT eHEALTH checklist (V 1.6.1).

## Abbreviations

AMR: antimicrobial resistance
CONSORT: Consolidated Standards of Reporting Trials
KAP: knowledge of, attitude toward, and perceptions
NPC: nonplayer character
RCT: randomized controlled trial

## Data Availability

Deidentified data that support the findings of this study are available on request from AC or ZH.
