# Supplementary material for: An Evidence-Based Serious Game App for Public Education on Antibiotic Use and Resistance: Randomized Controlled Trial
Source: JMIR Serious Games. 2024 Sep 5;12:e59848. doi: 10.2196/59848 (PMC11413539; doi:10.2196/59848)
Supplement: Multimedia Appendix 1 [file games_v12i1e59848_app1.docx]

**Table S1. List of bite-sized messages released by the six non-player characters (NPCs) in the “SteWARdS Antibiotic Defence” app.**

| **In-app NPC placeholder** | **World** | **NPC messages** |
| --- | --- | --- |
| Little girl | Supermarket | Sharing antibiotics with others, even if you experience the same symptoms, is not advisable. One should avoid self-medication using leftover antibiotics. |
| Supermarket bulletin board |  | Antibiotics resistance can affect anyone, of any age, from any country, as resistant bacteria can spread from person to person. |
| Little boy | Park | Not finishing the prescribed course of antibiotics encourages the development of antibiotic-resistant bacteria. |
| Park bulletin board |  | Antibiotic-resistance causes medical procedures to become more dangerous, leading to longer hospital stays, higher medical costs, and increased deaths. |
| Girl | Train station | Not all illnesses are the same even if they cause the same symptoms. Do not request for the same antibiotics even if they have helped you get better previously. |
| Train station bulletin board |  | Misuse and overuse of antibiotics is one of the main causes of antibiotic resistance. Antibiotic resistance is rising to dangerously high levels in all parts of the world. |

**Table S2. Knowledge, Attitudes, and Perception questions pre and post intervention.**

| **Qn.** | **Knowledge questions** | **Response options** |
| --- | --- | --- |
| 1 | If bacteria are resistant to antibiotics, it can be very difficult or impossible to treat the infections they cause. | True/False |
| 2 | It is okay to buy the same antibiotics or request for them from a doctor, if they had helped you get better previously when you had the same symptoms. |  |
| 3 | It is okay to use antibiotics that were given to a friend or family member, as long as they were used to treat the same illness. |  |
| 4 | I can stop my antibiotics course when I start feeling better. |  |
| 5 | Antibiotic resistance is an issue that could affect me or my family. |  |
| 6 | Antibiotic resistance is only a problem for people who take antibiotics regularly. |  |
| 7 | Many infections are becoming increasingly resistant to antibiotics treatment. |  |
| 8 | Antibiotic resistance is an issue in other countries and in Singapore. |  |
| 9 | Antibiotic resistance occurs when your body becomes resistant to antibiotics and antibiotics no longer work as well. |  |
| 10 | Bacteria which are resistant to antibiotics can spread from person to person. |  |
| 11 | Antibiotic-resistant infections could make medical procedures like surgery, organ transplant, and cancer treatment much more dangerous. |  |
|  | **Statements on attitudes and perceptions** | **Response options** |
| 12 | I feel that there is no harm in taking antibiotics. | Agree/ Disagree/ Unsure |
| 13 | I need antibiotics to help me to recover faster from the common cold and flu. |  |
| 14 | I need antibiotics to help me to recover from serious symptoms of the common cold and flu. |  |
| 15 | I need antibiotics if I continue to have flu symptoms after two weeks. |  |
| 16 | I will keep leftover antibiotics for future use if I have similar symptoms. |  |
| 17 | I would stop my course of antibiotics if I am concurrently using alternative remedy (e.g. Traditional Chinese Medicine, Ayurveda medicine, JAMU). |  |
| 18 | I am scared of getting antibiotic-resistant infections. |  |
| 19 | I will see another doctor if my doctor does not give me antibiotics. |  |
| 20 | How I use antibiotics does not affect my chances of getting antibiotic-resistant infections |  |
| 21 | How I use antibiotics does not affect other people’s chance of getting antibiotic resistant infections. |  |
| 22 | I normally keep antibiotic stocks at home in case of emergency. |  |
| 23 | If my family member is sick, I will usually give my antibiotics to them. |  |
| 24 | I normally stop taking antibiotics when I start feeling better. |  |
| 25 | I will take leftover antibiotics when I think I need them. |  |

Some knowledge questions are reversed scored at post-intervention to reduce the learning effect.

**
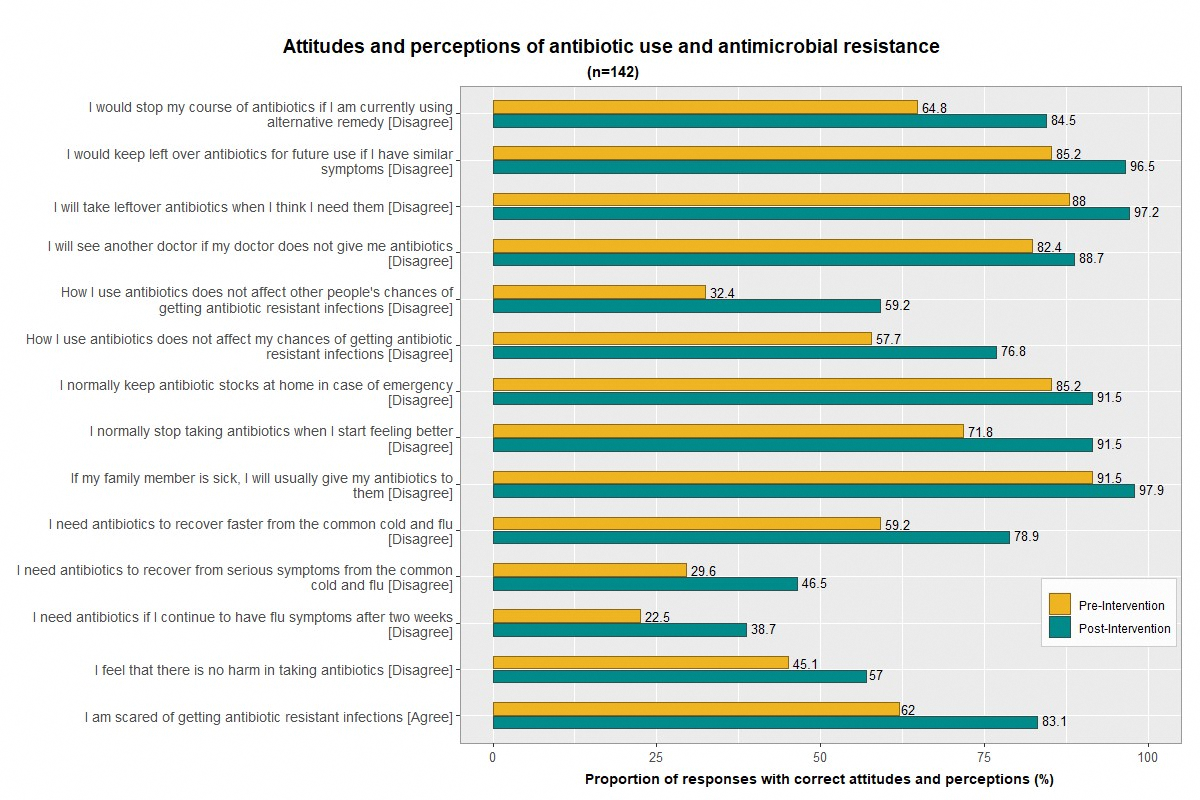

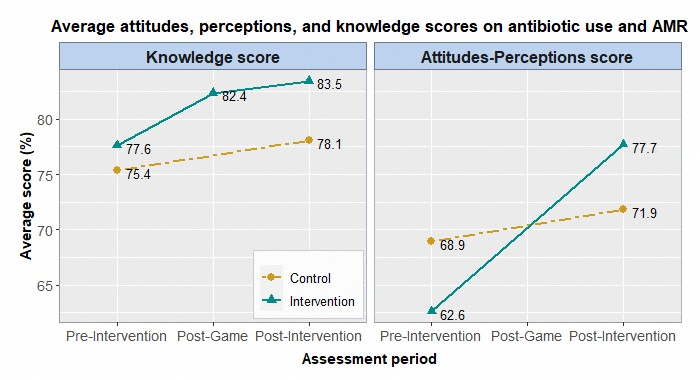
**

**Figure S1.** Mean standardized Knowledge and Attitudes-Perceptions score at baseline and post-intervention.

**Figure S2.** Proportion of correct attitudes-perception scores at baseline and post-intervention.

**Table S3. Reasons participants would or would not recommend “SteWARdS Antibiotic Defence” to others.**

| **Themes** | **Subthemes** | **Sample Quotes** |
| --- | --- | --- |
| Acceptability of the serious-game app | (+) Participants found the app enjoyable and interesting.  (n = 50/121*) | “Because it is an interesting way to learn about the use of antibiotics and antibiotics resistance. (Play & learn concept)”  “It is enjoyable to play the game.” |
|  | (+) Participants felt engaged during gameplay.  (n = 17/121) | “The app is interactive and engaging.”  “[learning through the app] is much more engaging compared to reading or through long videos.” |
|  | (-) Some participants felt that the app was uninteresting.  (n =4/21*) | “Too boring.”  “The game gets repetitive after a while.” |
| Value in learning concepts through the serious-game app | (+) Participants felt that the app is educational and raises awareness on antibiotic use and resistance.  (n = 77/121) | “…increases one's knowledge about antibiotic resistance.”  “[the app is] simultaneously fruitful in fulfilling its objectives of increasing the public's awareness of antibiotics.” |
|  | (+) Participants felt that the information disseminated through the app was important or useful to know.  (n = 11/121) | “It is important to learn about antibiotic resistance and its problems.”  “Most people especially [the] elderly would not know how to manage antibiotics the correct way.” |
| Usability of the serious-game app | (+) The information and concepts in the app were easy to understand.  (n = 17/171) | “[It was] easy to play and understand the concepts.”  “It provides information about antibiotic resistance in an easy to understand, engaging way.” |
|  | (-) Some participants were frustrated with difficulties in navigating the app.  (n = 8/21) | “Loading and game play was a little slow.”  “There were technical issues that made completing the game annoying.” |
|  | (-) Some participants felt that the mini-games were difficult to play.  (n = 3/21) | “The games—Tower Defence and Endless Runner—are difficult to play. Users might get discouraged and not complete the game.”  “Seniors may not be able to understand how to play the games.” |
|  | (-) Poor user experience  (n = 6/21) | “The user experience is not up to [the] standard gaming [experience].”  “[in-app text is too] small to read the information.” |

*121 participants would recommend the app to others and 21 participants would not.

**Table S4. Participants’ suggestions to improve the “SteWARdS antibiotic defence” app.**

| **Improvement** | **Summary** |
| --- | --- |
| Refine game mechanics  (Rules, game goals, actions, and strategies etc.) | - The mini games should reinforce concepts that the study team intended to teach. For e.g., Match3 (mini-game) does not teach about antibiotics but leans towards self-care. - Some participants wanted more mini-games while some preferred the app to focus on one in-depth game. - Reduce repetitiveness and duration of gameplay. - Increase the number of interactive elements in the game. - Inform how games are related to each other (e.g., Tower Defence and Endless Runner) - Increase the difficulty of higher levels. For e.g., level 9’s difficulty should not be the easiest in Tower Defence. - Calibrate the in-game economy to optimize the game’s difficulty level. - Making the player character jump between worlds and walk to specific NPCs to access the mini-games is tedious and unnecessary. - One participant suggested a drag-and-drop function for building turrets in Tower Defence. - Improve instructional clarity. - Movement controls should be more user friendly. - Review the wordings. One participant mentioned that not everyone will understand the term “NPC” and suggested using “character” instead. |
| Enhance user interface (UI)/ aesthetics  (The space where interactions occur – e.g., screens, appearances) | - Improve the graphics in the mini games. - Follow a consistent art style. - Enlarge the words in the text boxes and enable a read aloud option. - Distinguish the interactable Non-Player Characters from the background. - Improve the graphics so that the enemies resemble bacteria and viruses more closely. - Improve the intuitiveness of the user interface for older people to navigate the game easily. |
| More app features | - Add music and sound effects within the mini games. - The games in the app should be simple and appeal to people of all ages. - Allow users to create personal profiles. - Include in-app game demonstrations. |
| Bug fixes | - Improve the app’s responsiveness, sensitivity, and speed. - Fix the glitches and typo errors in the app. |
