# Supplementary material for: An Evidence-Based Serious Game App for Public Education on Antibiotic Use and Resistance: Randomized Controlled Trial
Source: JMIR Serious Games. 2024 Sep 5;12:e59848. doi: 10.2196/59848 (PMC11413539; doi:10.2196/59848)
Supplement: Multimedia Appendix 2 [file games_v12i1e59848_app2.pdf]

CONSORT-EHEALTH (V 1.6.1) - Submission/Publication Form

The CONSORT-EHEALTH checklist is intended for authors of randomized trials evaluating web-based and Internet-based applications/interventions, including mobile interventions, electronic games (incl multiplayer games), social media, certain telehealth applications, and other interactive and/or networked electronic applications. Some of the items (e.g. all subitems under item 5 - description of the intervention) may also be applicable for other study designs.

The goal of the CONSORT EHEALTH checklist and guideline is to be
a) a guide for reporting for authors of RCTs,
b) to form a basis for appraisal of an ehealth trial (in terms of validity)

CONSORT-EHEALTH items/subitems are MANDATORY reporting items for studies published in the Journal of Medical Internet Research and other journals / scientific societies endorsing the checklist.

Items numbered 1., 2., 3., 4a., 4b etc are original CONSORT or CONSORT-NPT (non-pharmacologic treatment) items.
Items with Roman numerals (i., ii, iii, iv etc.) are CONSORT-EHEALTH extensions/clarifications.

As the CONSORT-EHEALTH checklist is still considered in a formative stage, we would ask that you also RATE ON A SCALE OF 1-5 how important/useful you feel each item is FOR THE PURPOSE OF THE CHECKLIST and reporting guideline (optional).

Mandatory reporting items are marked with a red *.
In the textboxes, either copy & paste the relevant sections from your manuscript into this form - please include any quotes from your manuscript in QUOTATION MARKS,
or answer directly by providing additional information not in the manuscript, or elaborating on why the item was not relevant for this study.

YOUR ANSWERS WILL BE PUBLISHED AS A SUPPLEMENTARY FILE TO YOUR PUBLICATION IN JMIR AND ARE CONSIDERED PART OF YOUR PUBLICATION (IF ACCEPTED).
Please fill in these questions diligently. Information will not be copyedited, so please use proper spelling and grammar, use correct capitalization, and avoid abbreviations.

DO NOT FORGET TO SAVE AS PDF _AND_ CLICK THE SUBMIT BUTTON SO YOUR ANSWERS ARE IN OUR DATABASE !!!

Citation Suggestion (if you append the pdf as Appendix we suggest to cite this paper in the caption):
Eysenbach G, CONSORT-EHEALTH Group

CONSORT-EHEALTH: Improving and Standardizing Evaluation Reports of Web-based and Mobile Health Interventions
J Med Internet Res 2011;13(4):e126
URL: http://www.jmir.org/2011/4/e126/
doi: 10.2196/jmir.1923
PMID: 22209829

ojingtengjt@gmail.com Switch accounts

Not shared

Draft saved

* Indicates required question

Your name *

First Last

Zhilian Huang

Primary Affiliation (short), City, Country *

University of Toronto, Toronto, Canada

Tan Tock Seng Hospital, Singapore, Singapore

Your e-mail address *

abc@gmail.com

zhilian_huang@ttsh.com.sg

Title of your manuscript *

Provide the (draft) title of your manuscript.

An evidence-based serious game app for public education on antibiotic use and resistance: A randomized controlled trial

Name of your App/Software/Intervention *

If there is a short and a long/alternate name, write the short name first and add the long name in brackets.

SteWARdS Antibiotic Defence

Evaluated Version (if any)

e.g. "V1", "Release 2017-03-01", "Version 2.0.27913"

Your answer

Language(s) *

What language is the intervention/app in? If multiple languages are available, separate by comma (e.g. "English, French")

English

URL of your Intervention Website or App

e.g. a direct link to the mobile app on app in appstore (itunes, Google Play), or URL of the website. If the intervention is a DVD or hardware, you can also link to an Amazon page.

Your answer

URL of an image/screenshot (optional)

Your answer

Accessibility *

Can an enduser access the intervention presently?

- ( ) access is free and open
- ( ) access only for special usergroups, not open
- ( ) access is open to everyone, but requires payment/subscription/in-app purchases
- (•) app/intervention no longer accessible
- ( ) Other:

Primary Medical Indication/Disease/Condition *

e.g. "Stress", "Diabetes", or define the target group in brackets after the condition, e.g. "Autism (Parents of children with)", "Alzheimers (Informal Caregivers of)"

Antimicrobial Resistance

Primary Outcomes measured in trial *

comma-separated list of primary outcomes reported in the trial

Knowledge, Attitudes, Perceptions

Secondary/other outcomes

Are there any other outcomes the intervention is expected to affect?

Level of User Satisfaction

Recommended "Dose" *

What do the instructions for users say on how often the app should be used?

- ( ) Approximately Daily
- ( ) Approximately Weekly
- ( ) Approximately Monthly
- ( ) Approximately Yearly
- (•) "as needed"
- ( ) Other:

Approx. Percentage of Users (starters) still using the app as recommended after 3 months *

- (•) unknown / not evaluated
- ( ) 0-10%
- ( ) 11-20%
- ( ) 21-30%
- ( ) 31-40%
- ( ) 41-50%
- ( ) 51-60%
- ( ) 61-70%
- ( ) 71%-80%
- ( ) 81-90%
- ( ) 91-100%
- ( ) Other:

Overall, was the app/intervention effective? *

- (selected) yes: all primary outcomes were significantly better in intervention group vs control
- partly: SOME primary outcomes were significantly better in intervention group vs control
- no statistically significant difference between control and intervention
- potentially harmful: control was significantly better than intervention in one or more outcomes
- inconclusive: more research is needed
- Other:

Article Preparation Status/Stage *

At which stage in your article preparation are you currently (at the time you fill in this form)

- not submitted yet - in early draft status
- not submitted yet - in late draft status, just before submission
- (selected) submitted to a journal but not reviewed yet
- submitted to a journal and after receiving initial reviewer comments
- submitted to a journal and accepted, but not published yet
- published
- Other:

Journal *

If you already know where you will submit this paper (or if it is already submitted), please provide the journal name (if it is not JMIR, provide the journal name under "other")

- ( ) not submitted yet / unclear where I will submit this
- ( ) Journal of Medical Internet Research (JMIR)
- ( ) JMIR mHealth and UHealth
- ( ) JMIR Serious Games
- ( ) JMIR Mental Health
- (•) JMIR Public Health
- ( ) JMIR Formative Research
- ( ) Other JMIR sister journal
- ( ) Other:

Is this a full powered effectiveness trial or a pilot/feasibility trial? *

- ( ) Pilot/feasibility
- ( ) Fully powered

Manuscript tracking number *

If this is a JMIR submission, please provide the manuscript tracking number under "other" (The ms tracking number can be found in the submission acknowledgement email, or when you login as author in JMIR. If the paper is already published in JMIR, then the ms tracking number is the four-digit number at the end of the DOI, to be found at the bottom of each published article in JMIR)

- ( ) no ms number (yet) / not (yet) submitted to / published in JMIR
- (•) Other: ms#59848

TITLE AND ABSTRACT

1a) TITLE: Identification as a randomized trial in the title

1a) Does your paper address CONSORT item 1a? *

I.e does the title contain the phrase "Randomized Controlled Trial"? (if not, explain the reason under "other")

- (●) yes
- ( ) Other:

1a-i) Identify the mode of delivery in the title

Identify the mode of delivery. Preferably use “web-based” and/or “mobile” and/or “electronic game” in the title. Avoid ambiguous terms like “online”, “virtual”, “interactive”. Use “Internet-based” only if Intervention includes non-web-based Internet components (e.g. email), use “computer-based” or “electronic” only if offline products are used. Use “virtual” only in the context of “virtual reality” (3-D worlds). Use “online” only in the context of “online support groups”. Complement or substitute product names with broader terms for the class of products (such as “mobile” or “smart phone” instead of “iphone”), especially if the application runs on different platforms.

	1	2	3	4	5	
subitem not at all important	( )	( )	( )	( )	(●)	essential

Clear selection

Does your paper address subitem 1a-i? *

Copy and paste relevant sections from manuscript title (include quotes in quotation marks "like this" to indicate direct quotes from your manuscript), or elaborate on this item by providing additional information not in the ms, or briefly explain why the item is not applicable/relevant for your study

"evidence-based serious-game app"

1a-ii) Non-web-based components or important co-interventions in title

Mention non-web-based components or important co-interventions in title, if any (e.g., “with telephone support”).

	1	2	3	4	5	
subitem not at all important	○	○	◉	○	○	essential

Clear selection

Does your paper address subitem 1a-ii?

Copy and paste relevant sections from manuscript title (include quotes in quotation marks "like this" to indicate direct quotes from your manuscript), or elaborate on this item by providing additional information not in the ms, or briefly explain why the item is not applicable/relevant for your study

NA, there are no non-web-based components or other co-interventions.

1a-iii) Primary condition or target group in the title

Mention primary condition or target group in the title, if any (e.g., “for children with Type I Diabetes”) Example: A Web-based and Mobile Intervention with Telephone Support for Children with Type I Diabetes: Randomized Controlled Trial

	1	2	3	4	5	
subitem not at all important	○	○	○	○	◉	essential

Clear selection

Does your paper address subitem 1a-iii? *

Copy and paste relevant sections from manuscript title (include quotes in quotation marks "like this" to indicate direct quotes from your manuscript), or elaborate on this item by providing additional information not in the ms, or briefly explain why the item is not applicable/relevant for your study

"antibiotic use and resistance"

1b) ABSTRACT: Structured summary of trial design, methods, results, and conclusions

NPT extension: Description of experimental treatment, comparator, care providers, centers, and blinding status.

1b-i) Key features/functionalities/components of the intervention and comparator in the METHODS section of the ABSTRACT

Mention key features/functionalities/components of the intervention and comparator in the abstract. If possible, also mention theories and principles used for designing the site. Keep in mind the needs of systematic reviewers and indexers by including important synonyms. (Note: Only report in the abstract what the main paper is reporting. If this information is missing from the main body of text, consider adding it)

	1	2	3	4	5	
subitem not at all important	○	○	○	○	◉	essential

Clear selection

Does your paper address subitem 1b-i? *

Copy and paste relevant sections from the manuscript abstract (include quotes in quotation marks "like this" to indicate direct quotes from your manuscript), or elaborate on this item by providing additional information not in the ms, or briefly explain why the item is not applicable/relevant for your study

NA as there is already a protocol published in JMIR Research Protocols detailing these key features (with images).

1b-ii) Level of human involvement in the METHODS section of the ABSTRACT

Clarify the level of human involvement in the abstract, e.g., use phrases like “fully automated” vs. “therapist/nurse/care provider/physician-assisted” (mention number and expertise of providers involved, if any). (Note: Only report in the abstract what the main paper is reporting. If this information is missing from the main body of text, consider adding it)

	1	2	3	4	5	
subitem not at all important	○	○	◉	○	○	essential

Clear selection

Does your paper address subitem 1b-ii?

Copy and paste relevant sections from the manuscript abstract (include quotes in quotation marks "like this" to indicate direct quotes from your manuscript), or elaborate on this item by providing additional information not in the ms, or briefly explain why the item is not applicable/relevant for your study

NA, after being briefly taught how to use the app, users play the game at their own time without assistance (unless requested for).

1b-iii) Open vs. closed, web-based (self-assessment) vs. face-to-face assessments in the METHODS section of the ABSTRACT

Mention how participants were recruited (online vs. offline), e.g., from an open access website or from a clinic or a closed online user group (closed usergroup trial), and clarify if this was a purely web-based trial, or there were face-to-face components (as part of the intervention or for assessment). Clearly say if outcomes were self-assessed through questionnaires (as common in web-based trials). Note: In traditional offline trials, an open trial (open-label trial) is a type of clinical trial in which both the researchers and participants know which treatment is being administered. To avoid confusion, use “blinded” or “unblinded” to indicated the level of blinding instead of “open”, as “open” in web-based trials usually refers to “open access” (i.e. participants can self-enrol). (Note: Only report in the abstract what the main paper is reporting. If this information is missing from the main body of text, consider adding it)

	1	2	3	4	5	
subitem not at all important	○	○	○	○	●	essential

Clear selection

Does your paper address subitem 1b-iii?

Copy and paste relevant sections from the manuscript abstract (include quotes in quotation marks "like this" to indicate direct quotes from your manuscript), or elaborate on this item by providing additional information not in the ms, or briefly explain why the item is not applicable/relevant for your study

Participants were recruited offline - "recruiting visitors 18–65 years old from two polyclinics in Singapore".

Outcomes were self-assessed - "Knowledge questions on antibiotic use and AMR (11 binary questions) was self-administered at baseline, immediately after the intervention, and 6–10 weeks post-intervention, while attitudes and perception questions (14 three-point Likert scale questions) were self-administered at baseline and 6–10 weeks post-intervention."

1b-iv) RESULTS section in abstract must contain use data

Report number of participants enrolled/assessed in each group, the use/uptake of the intervention (e.g., attrition/adherence metrics, use over time, number of logins etc.), in addition to primary/secondary outcomes. (Note: Only report in the abstract what the main paper is reporting. If this information is missing from the main body of text, consider adding it)

	1	2	3	4	5	
subitem not at all important	○	○	○	○	◉	essential

Clear selection

Does your paper address subitem 1b-iv?

Copy and paste relevant sections from the manuscript abstract (include quotes in quotation marks "like this" to indicate direct quotes from your manuscript), or elaborate on this item by providing additional information not in the ms, or briefly explain why the item is not applicable/relevant for your study

Number of participants - "Participants (n=348; 142 Intervention, 206 Controls) "

Primary outcomes - "Intervention group participants showed significantly greater improvement in knowledge scores compared with controls [0.58 (95% CI: 0.28, 0.87)] after accounting for age, educational level, and exposure to advertisements on antibiotics and AMR. Intervention participants also showed significantly greater improvement in attitude-perception score [0.98 (0.44, 1.52)] after adjusting for marital status and race. "

1b-v) CONCLUSIONS/DISCUSSION in abstract for negative trials

Conclusions/Discussions in abstract for negative trials: Discuss the primary outcome - if the trial is negative (primary outcome not changed), and the intervention was not used, discuss whether negative results are attributable to lack of uptake and discuss reasons. (Note: Only report in the abstract what the main paper is reporting. If this information is missing from the main body of text, consider adding it)

	1	2	3	4	5	
subitem not at all important	◉	○	○	○	○	essential

Clear selection

Does your paper address subitem 1b-v?

Copy and paste relevant sections from the manuscript abstract (include quotes in quotation marks "like this" to indicate direct quotes from your manuscript), or elaborate on this item by providing additional information not in the ms, or briefly explain why the item is not applicable/relevant for your study

NA, changes in primary outcomes were observed.

INTRODUCTION

2a) In INTRODUCTION: Scientific background and explanation of rationale

2a-i) Problem and the type of system/solution

Describe the problem and the type of system/solution that is object of the study: intended as stand-alone intervention vs. incorporated in broader health care program? Intended for a particular patient population? Goals of the intervention, e.g., being more cost-effective to other interventions, replace or complement other solutions? (Note: Details about the intervention are provided in “Methods” under 5)

	1	2	3	4	5	
subitem not at all important	○	○	○	○	◉	essential

Clear selection

Does your paper address subitem 2a-i? *

Copy and paste relevant sections from the manuscript (include quotes in quotation marks "like this" to indicate direct quotes from your manuscript), or elaborate on this item by providing additional information not in the ms, or briefly explain why the item is not applicable/relevant for your study

Problem - "...but various national educational campaigns on antibiotics and AMR, including those in Singapore, have shown ambiguous effectiveness. Despite yearly public outreach and multiple national campaigns, misconceptions about antibiotics – such as believing that antibiotics treat viral infections – continue to persist among the Singaporean population".

Goals of the intervention - "to explore novel public education methods beyond traditional modalities (e.g., posters and pamphlets)", "to deliver adult education on appropriate antibiotic use and AMR"

2a-ii) Scientific background, rationale: What is known about the (type of) system

Scientific background, rationale: What is known about the (type of) system that is the object of the study (be sure to discuss the use of similar systems for other conditions/diagnoses, if appropiate), motivation for the study, i.e. what are the reasons for and what is the context for this specific study, from which stakeholder viewpoint is the study performed, potential impact of findings [2]. Briefly justify the choice of the comparator.

	1	2	3	4	5	
subitem not at all important	○	○	○	○	◉	essential

Clear selection

Does your paper address subitem 2a-ii? *

Copy and paste relevant sections from the manuscript (include quotes in quotation marks "like this" to indicate direct quotes from your manuscript), or elaborate on this item by providing additional information not in the ms, or briefly explain why the item is not applicable/relevant for your study

What is known - "Preliminary evidence of serious-games interventions on antibiotic use and AMR has shown promise in improving the knowledge of appropriate antibiotic use"

Motivation of the study - "However, these studies were primarily focused on children and students. Adults have greater access to antibiotics and are more likely to pass down antibiotic use behaviours to the younger generation. Hence, it is essential to educate young adults on appropriate antibiotic use. These studies also limited participants to a controlled environment with restricted playing time and may not fully capture real-world behaviour. Furthermore, many studies overlooked the importance of long-term knowledge retention. The paucity of high-quality randomized controlled trials (RCTs) in the real-world setting highlights a pertinent gap in the evidence base"

2b) In INTRODUCTION: Specific objectives or hypotheses

Does your paper address CONSORT subitem 2b? *

Copy and paste relevant sections from the manuscript (include quotes in quotation marks "like this" to indicate direct quotes from your manuscript), or elaborate on this item by providing additional information not in the ms, or briefly explain why the item is not applicable/relevant for your study

Objectives - "We developed an evidence-based serious-game app—"SteWARdS Antibiotic Defence"—to deliver adult education on appropriate antibiotic use and AMR and aimed to evaluate its effectiveness in improving knowledge of, attitude toward, and perceptions (KAP) of antibiotic use and AMR in Singapore. We also sought user feedback on the "SteWARdS Antibiotic Defence" app"

METHODS

3a) Description of trial design (such as parallel, factorial) including allocation ratio

Does your paper address CONSORT subitem 3a? *

Copy and paste relevant sections from the manuscript (include quotes in quotation marks "like this" to indicate direct quotes from your manuscript), or elaborate on this item by providing additional information not in the ms, or briefly explain why the item is not applicable/relevant for your study

Trial design - "a parallel two-arm RCT with a 6–10-week follow-up period"

For more information, please kindly refer to the published protocol on JMIR Research Protocols - "An Evidence-Based Serious Game App for Public Education on Antibiotic Use and Antimicrobial Resistance: Protocol of a Randomized Controlled Trial"

3b) Important changes to methods after trial commencement (such as eligibility criteria), with reasons

Does your paper address CONSORT subitem 3b? *

Copy and paste relevant sections from the manuscript (include quotes in quotation marks "like this" to indicate direct quotes from your manuscript), or elaborate on this item by providing additional information not in the ms, or briefly explain why the item is not applicable/relevant for your study

NA, no changes were made to the methods after trial commencement.

3b-i) Bug fixes, Downtimes, Content Changes

Bug fixes, Downtimes, Content Changes: ehealth systems are often dynamic systems. A description of changes to methods therefore also includes important changes made on the intervention or comparator during the trial (e.g., major bug fixes or changes in the functionality or content) (5-iii) and other “unexpected events” that may have influenced study design such as staff changes, system failures/downtimes, etc. [2].

	1	2	3	4	5	
subitem not at all important	○	○	●	○	○	essential

Clear selection

Does your paper address subitem 3b-i?

Copy and paste relevant sections from the manuscript (include quotes in quotation marks "like this" to indicate direct quotes from your manuscript), or elaborate on this item by providing additional information not in the ms, or briefly explain why the item is not applicable/relevant for your study

NA, no changes were made to the intervention (i.e. the game app) during the trial.

4a) Eligibility criteria for participants

Does your paper address CONSORT subitem 4a? *

Copy and paste relevant sections from the manuscript (include quotes in quotation marks "like this" to indicate direct quotes from your manuscript), or elaborate on this item by providing additional information not in the ms, or briefly explain why the item is not applicable/relevant for your study

Exclusion criteria - "We excluded those with visual/cognitive impairment, not proficient with smartphone apps, and those unable to install Android mobile applications from the study."

For the inclusion criteria, please kindly refer to the published protocol on JMIR Research Protocols - "An Evidence-Based Serious Game App for Public Education on Antibiotic Use and Antimicrobial Resistance: Protocol of a Randomized Controlled Trial"

4a-i) Computer / Internet literacy

Computer / Internet literacy is often an implicit "de facto" eligibility criterion - this should be explicitly clarified.

	1	2	3	4	5	
subitem not at all important	○	○	◉	○	○	essential

Clear selection

Does your paper address subitem 4a-i?

Copy and paste relevant sections from the manuscript (include quotes in quotation marks "like this" to indicate direct quotes from your manuscript), or elaborate on this item by providing additional information not in the ms, or briefly explain why the item is not applicable/relevant for your study

Exclusion criteria - "We excluded those with visual/cognitive impairment, not proficient with smartphone apps, and those unable to install Android mobile applications from the study"

4a-ii) Open vs. closed, web-based vs. face-to-face assessments:

Open vs. closed, web-based vs. face-to-face assessments: Mention how participants were recruited (online vs. offline), e.g., from an open access website or from a clinic, and clarify if this was a purely web-based trial, or there were face-to-face components (as part of the intervention or for assessment), i.e., to what degree got the study team to know the participant. In online-only trials, clarify if participants were quasi-anonymous and whether having multiple identities was possible or whether technical or logistical measures (e.g., cookies, email confirmation, phone calls) were used to detect/prevent these.

	1	2	3	4	5	
subitem not at all important	○	○	○	○	◉	essential

Clear selection

Does your paper address subitem 4a-ii? *

Copy and paste relevant sections from the manuscript (include quotes in quotation marks "like this" to indicate direct quotes from your manuscript), or elaborate on this item by providing additional information not in the ms, or briefly explain why the item is not applicable/relevant for your study

Participants were recruited offline face-to-face - "We recruited patients and their caregivers from two government-funded primary care clinics (i.e., polyclinics) in Singapore.", "Trained data collectors were employed to recruit participants in the Polyclinics. The data collectors first approach potential participants to explain the study before obtaining their informed consent to participate in our study."

Extent to which study team knew the participants - "Apart from the data collectors, all other study team members (including the data analyst) were blinded to the group assignment."

4a-iii) Information giving during recruitment

Information given during recruitment. Specify how participants were briefed for recruitment and in the informed consent procedures (e.g., publish the informed consent documentation as appendix, see also item X26), as this information may have an effect on user self-selection, user expectation and may also bias results.

	1	2	3	4	5	
subitem not at all important	○	○	◉	○	○	essential

Clear selection

Does your paper address subitem 4a-iii?

Copy and paste relevant sections from the manuscript (include quotes in quotation marks "like this" to indicate direct quotes from your manuscript), or elaborate on this item by providing additional information not in the ms, or briefly explain why the item is not applicable/relevant for your study

NA, as details are already provided in a published protocol in JMIR Research Protocols - "An Evidence-Based Serious Game App for Public Education on Antibiotic Use and Antimicrobial Resistance: Protocol of a Randomized Controlled Trial"

4b) Settings and locations where the data were collected

Does your paper address CONSORT subitem 4b? *

Copy and paste relevant sections from the manuscript (include quotes in quotation marks "like this" to indicate direct quotes from your manuscript), or elaborate on this item by providing additional information not in the ms, or briefly explain why the item is not applicable/relevant for your study

Location of recruitment and baseline survey - "We recruited patients and their caregivers from two government-funded primary care clinics (i.e., polyclinics) in Singapore."

Subsequent surveys were conducted online - Control Group: "A study team member (i.e., data collector) sends participants a detailed WhatsApp message six weeks after their recruitment date to inform them to complete the post-intervention KAP questionnaire. ", Intervention Group: "Participants had to complete the “SteWARdS Antibiotic Defence” game quest and satisfaction survey and inform the study team via WhatsApp upon intervention completion. Six weeks after participants complete the intervention, a study team member sends a detailed WhatsApp message to inform them to complete the post-intervention KAP questionnaire."

4b-i) Report if outcomes were (self-)assessed through online questionnaires

Clearly report if outcomes were (self-)assessed through online questionnaires (as common in web-based trials) or otherwise.

	1	2	3	4	5	
subitem not at all important	○	○	○	○	◉	essential

Clear selection

Does your paper address subitem 4b-i? *

Copy and paste relevant sections from the manuscript (include quotes in quotation marks "like this" to indicate direct quotes from your manuscript), or elaborate on this item by providing additional information not in the ms, or briefly explain why the item is not applicable/relevant for your study

Outcomes were self-assessed - "Consented participants were randomized and asked to complete the baseline questionnaire, which collected the KAP responses and participants' demographic data.", "A study team member (i.e., data collector) sends participants a detailed WhatsApp message six weeks after their recruitment date to inform them to complete the post-intervention KAP questionnaire.", "Participants had to complete the "SteWARdS Antibiotic Defence" game quest and satisfaction survey and inform the study team via WhatsApp upon intervention completion. Six weeks after participants complete the intervention, a study team member sends a detailed WhatsApp message to inform them to complete the post-intervention KAP questionnaire."

For details on the questionnaire, please kindly refer to the published protocol on JMIR Research Protocols - "An Evidence-Based Serious Game App for Public Education on Antibiotic Use and Antimicrobial Resistance: Protocol of a Randomized Controlled Trial"

4b-ii) Report how institutional affiliations are displayed

Report how institutional affiliations are displayed to potential participants [on ehealth media], as affiliations with prestigious hospitals or universities may affect volunteer rates, use, and reactions with regards to an intervention.(Not a required item – describe only if this may bias results)

	1	2	3	4	5	
subitem not at all important	○	○	○	○	○	essential

Does your paper address subitem 4b-ii?

Copy and paste relevant sections from the manuscript (include quotes in quotation marks "like this" to indicate direct quotes from your manuscript), or elaborate on this item by providing additional information not in the ms, or briefly explain why the item is not applicable/relevant for your study

Your answer

5) The interventions for each group with sufficient details to allow replication, including how and when they were actually administered

5-i) Mention names, credential, affiliations of the developers, sponsors, and owners

Mention names, credential, affiliations of the developers, sponsors, and owners [6] (if authors/evaluators are owners or developer of the software, this needs to be declared in a "Conflict of interest" section or mentioned elsewhere in the manuscript).

	1	2	3	4	5	
subitem not at all important	○	○	◉	○	○	essential

Clear selection

Does your paper address subitem 5-i?

Copy and paste relevant sections from the manuscript (include quotes in quotation marks "like this" to indicate direct quotes from your manuscript), or elaborate on this item by providing additional information not in the ms, or briefly explain why the item is not applicable/relevant for your study

Acknowledgement of app co-developers - ". We would also like to thank the staff and student interns from Temasek Polytechnic's School of Engineering and School of Informatics & IT for their contribution in maintaining the "SteWARdS antibiotic Defence" app and database during the study."

For more details, please kindly refer to the published protocol on JMIR Research Protocols - "An Evidence-Based Serious Game App for Public Education on Antibiotic Use and Antimicrobial Resistance: Protocol of a Randomized Controlled Trial"

5-ii) Describe the history/development process

Describe the history/development process of the application and previous formative evaluations (e.g., focus groups, usability testing), as these will have an impact on adoption/use rates and help with interpreting results.

	1	2	3	4	5	
subitem not at all important	○	○	◉	○	○	essential

Clear selection

Does your paper address subitem 5-ii?

Copy and paste relevant sections from the manuscript (include quotes in quotation marks "like this" to indicate direct quotes from your manuscript), or elaborate on this item by providing additional information not in the ms, or briefly explain why the item is not applicable/relevant for your study

The mobile game app took over a year to develop, with multiple rounds of usability testing within and outside of the study team.

5-iii) Revisions and updating

Revisions and updating. Clearly mention the date and/or version number of the application/intervention (and comparator, if applicable) evaluated, or describe whether the intervention underwent major changes during the evaluation process, or whether the development and/or content was “frozen” during the trial. Describe dynamic components such as news feeds or changing content which may have an impact on the replicability of the intervention (for unexpected events see item 3b).

	1	2	3	4	5	
subitem not at all important	○	○	◉	○	○	essential

Clear selection

Does your paper address subitem 5-iii?

Copy and paste relevant sections from the manuscript (include quotes in quotation marks "like this" to indicate direct quotes from your manuscript), or elaborate on this item by providing additional information not in the ms, or briefly explain why the item is not applicable/relevant for your study

NA, the intervention (i.e. the mobile game app) did not undergo major changes during the evaluation process. Content did not change throughout the trial and there are no impacts on replicability.

5-iv) Quality assurance methods

Provide information on quality assurance methods to ensure accuracy and quality of information provided [1], if applicable.

	1	2	3	4	5	
subitem not at all important	○	○	○	○	○	essential

Does your paper address subitem 5-iv?

Copy and paste relevant sections from the manuscript (include quotes in quotation marks "like this" to indicate direct quotes from your manuscript), or elaborate on this item by providing additional information not in the ms, or briefly explain why the item is not applicable/relevant for your study

Your answer

5-v) Ensure replicability by publishing the source code, and/or providing screenshots/screen-capture video, and/or providing flowcharts of the algorithms used

Ensure replicability by publishing the source code, and/or providing screenshots/screen-capture video, and/or providing flowcharts of the algorithms used. Replicability (i.e., other researchers should in principle be able to replicate the study) is a hallmark of scientific reporting.

	1	2	3	4	5	
subitem not at all important	○	○	◉	○	○	essential

Clear selection

Does your paper address subitem 5-v?

Copy and paste relevant sections from the manuscript (include quotes in quotation marks "like this" to indicate direct quotes from your manuscript), or elaborate on this item by providing additional information not in the ms, or briefly explain why the item is not applicable/relevant for your study

NA, screenshots of the game are not provided in this manuscript but can be found in the published protocol, along with descriptions of the game, on JMIR Research Protocols - "An Evidence-Based Serious Game App for Public Education on Antibiotic Use and Antimicrobial Resistance: Protocol of a Randomized Controlled Trial"

5-vi) Digital preservation

Digital preservation: Provide the URL of the application, but as the intervention is likely to change or disappear over the course of the years; also make sure the intervention is archived (Internet Archive, webcitation.org, and/or publishing the source code or screenshots/videos alongside the article). As pages behind login screens cannot be archived, consider creating demo pages which are accessible without login.

	1	2	3	4	5	
subitem not at all important	○	○	◉	○	○	essential

Clear selection

Does your paper address subitem 5-vi?

Copy and paste relevant sections from the manuscript (include quotes in quotation marks "like this" to indicate direct quotes from your manuscript), or elaborate on this item by providing additional information not in the ms, or briefly explain why the item is not applicable/relevant for your study

NA, the app is available on mobile only and hence does not have a URL. For screenshots of the game app, please kindly refer to the published protocol on JMIR Research Protocols - "An Evidence-Based Serious Game App for Public Education on Antibiotic Use and Antimicrobial Resistance: Protocol of a Randomized Controlled Trial"

5-vii) Access

Access: Describe how participants accessed the application, in what setting/context, if they had to pay (or were paid) or not, whether they had to be a member of specific group. If known, describe how participants obtained "access to the platform and Internet" [1]. To ensure access for editors/reviewers/readers, consider to provide a "backdoor" login account or demo mode for reviewers/readers to explore the application (also important for archiving purposes, see vi).

	1	2	3	4	5	
subitem not at all important	○	○	○	○	◉	essential

Clear selection

Does your paper address subitem 5-vii? *

Copy and paste relevant sections from the manuscript (include quotes in quotation marks "like this" to indicate direct quotes from your manuscript), or elaborate on this item by providing additional information not in the ms, or briefly explain why the item is not applicable/relevant for your study

Participants accessed the application by downloading the mobile game app - "Intervention group participants had to download the SteWARdS Antibiotic Defence app (only available in English and on the Android platform) on their smartphones and complete the quest in the app"

Participants were reimbursed for their time - "We reimbursed participants a small fee for completing the surveys and game quest."

As the app is no longer accessible, backdoor login would not be possible. However, screenshots and descriptions of the game are available on the published protocol on JMIR Research Protocols - "An Evidence-Based Serious Game App for Public Education on Antibiotic Use and Antimicrobial Resistance: Protocol of a Randomized Controlled Trial".

5-viii) Mode of delivery, features/functionalities/components of the intervention and comparator, and the theoretical framework

Describe mode of delivery, features/functionalities/components of the intervention and comparator, and the theoretical framework [6] used to design them (instructional strategy [1], behaviour change techniques, persuasive features, etc., see e.g., [7, 8] for terminology). This includes an in-depth description of the content (including where it is coming from and who developed it) [1],” whether [and how] it is tailored to individual circumstances and allows users to track their progress and receive feedback” [6]. This also includes a description of communication delivery channels and – if computer-mediated communication is a component – whether communication was synchronous or asynchronous [6]. It also includes information on presentation strategies [1], including page design principles, average amount of text on pages, presence of hyperlinks to other resources, etc. [1].

	1	2	3	4	5	
subitem not at all important	○	○	●	○	○	essential

Clear selection

Does your paper address subitem 5-viii? *

Copy and paste relevant sections from the manuscript (include quotes in quotation marks "like this" to indicate direct quotes from your manuscript), or elaborate on this item by providing additional information not in the ms, or briefly explain why the item is not applicable/relevant for your study

Mode of intervention delivery and comparatorn- "Participants in the intervention group had to download and complete the quest in the “SteWARdS Antibiotic Defence” app on their smartphones, while controls received no intervention. "

The theoretical framework used to develop the app is not described in this paper. It is, however, described on the published protocol on JMIR Research Protocols - "An Evidence-Based Serious Game App for Public Education on Antibiotic Use and Antimicrobial Resistance: Protocol of a Randomized Controlled Trial".

5-ix) Describe use parameters

Describe use parameters (e.g., intended “doses” and optimal timing for use). Clarify what instructions or recommendations were given to the user, e.g., regarding timing, frequency, heaviness of use, if any, or was the intervention used ad libitum.

	1	2	3	4	5	
subitem not at all important	○	○	◉	○	○	essential

Clear selection

Does your paper address subitem 5-ix?

Copy and paste relevant sections from the manuscript (include quotes in quotation marks "like this" to indicate direct quotes from your manuscript), or elaborate on this item by providing additional information not in the ms, or briefly explain why the item is not applicable/relevant for your study

NA, participants were not recommended any intended "doses" of usage and were allowed to complete the game quest on their own time as we were interested in looking in real-world user behaviours. However, for the purposes of the research study, participants who did not complete the game quest two weeks after recruitment were sent reminders - "A study team member reminded participants who did not complete the quest two weeks after recruitment".

5-x) Clarify the level of human involvement

Clarify the level of human involvement (care providers or health professionals, also technical assistance) in the e-intervention or as co-intervention (detail number and expertise of professionals involved, if any, as well as “type of assistance offered, the timing and frequency of the support, how it is initiated, and the medium by which the assistance is delivered”. It may be necessary to distinguish between the level of human involvement required for the trial, and the level of human involvement required for a routine application outside of a RCT setting (discuss under item 21 – generalizability).

	1	2	3	4	5	
subitem not at all important	○	○	◉	○	○	essential

Clear selection

Does your paper address subitem 5-x?

Copy and paste relevant sections from the manuscript (include quotes in quotation marks "like this" to indicate direct quotes from your manuscript), or elaborate on this item by providing additional information not in the ms, or briefly explain why the item is not applicable/relevant for your study

No active assistance or co-intervention were provided to participants, though participants were provided a helpline to contact if they faced technical difficulties - "There is also an in-app WhatsApp helpline for participants to contact the study team for technical difficulties."

5-xi) Report any prompts/reminders used

Report any prompts/reminders used: Clarify if there were prompts (letters, emails, phone calls, SMS) to use the application, what triggered them, frequency etc. It may be necessary to distinguish between the level of prompts/reminders required for the trial, and the level of prompts/reminders for a routine application outside of a RCT setting (discuss under item 21 – generalizability).

	1	2	3	4	5	
subitem not at all important	○	○	○	○	◉	essential

Clear selection

Does your paper address subitem 5-xi? *

Copy and paste relevant sections from the manuscript (include quotes in quotation marks "like this" to indicate direct quotes from your manuscript), or elaborate on this item by providing additional information not in the ms, or briefly explain why the item is not applicable/relevant for your study

Reminders were sent to participants - Control Group: "The study team member sent a reminder if participants did not complete the questionnaire two weeks after the first WhatsApp message.", Intervention Group: "A study team member reminded participants who did not complete the quest two weeks after recruitment and two weeks after sending the first KAP WhatsApp message. "

5-xii) Describe any co-interventions (incl. training/support)

Describe any co-interventions (incl. training/support): Clearly state any interventions that are provided in addition to the targeted eHealth intervention, as ehealth intervention may not be designed as stand-alone intervention. This includes training sessions and support [1]. It may be necessary to distinguish between the level of training required for the trial, and the level of training for a routine application outside of a RCT setting (discuss under item 21 – generalizability.

	1	2	3	4	5	
subitem not at all important	○	○	◉	○	○	essential

Clear selection

Does your paper address subitem 5-xii? *

Copy and paste relevant sections from the manuscript (include quotes in quotation marks "like this" to indicate direct quotes from your manuscript), or elaborate on this item by providing additional information not in the ms, or briefly explain why the item is not applicable/relevant for your study

NA, participants were briefly taught how to navigate the app at the point of recruitment but no further training was provided. Tutorials on how to play the game app were already built into the app.

6a) Completely defined pre-specified primary and secondary outcome measures, including how and when they were assessed

Does your paper address CONSORT subitem 6a? *

Copy and paste relevant sections from the manuscript (include quotes in quotation marks "like this" to indicate direct quotes from your manuscript), or elaborate on this item by providing additional information not in the ms, or briefly explain why the item is not applicable/relevant for your study

Primary outcomes - "The primary outcome of interest was the change in KAP score of antibiotic use and AMR. ", "Primary outcomes were assessed for the intervention and control groups at baseline, immediately post-intervention (intervention group only), and 6–10 weeks post-intervention (from baseline for controls) via self-administered questionnaires. Knowledge was measured via 11 True/False questions, while attitudes and perceptions were measured via 14 questions on a three-point Likert scale. Details of the KAP questionnaire are available in our published protocol"

Secondary outcomes - "The secondary outcome was the level of user satisfaction for the “SteWARdS Antibiotic Defence” app.", "For the intervention group, satisfaction with the “SteWARdS Antibiotic Defence” app was measured with four questions on a five-point Likert scale and the reason(s) for recommending/not recommending the app to others and suggestions for improvement were obtained via open-ended questions, upon quest completion."

6a-i) Online questionnaires: describe if they were validated for online use and apply CHERRIES items to describe how the questionnaires were designed/deployed

If outcomes were obtained through online questionnaires, describe if they were validated for online use and apply CHERRIES items to describe how the questionnaires were designed/deployed [9].

	1	2	3	4	5	
subitem not at all important	○	○	◉	○	○	essential

Clear selection

Does your paper address subitem 6a-i?

Copy and paste relevant sections from manuscript text

CHERRIES:
Design: Target Population - "Participants were 18 – 65 years old, had access to an Android smartphone, and were English literate"; Convenience Sampling - "The data collectors first approach potential participants to explain the study before obtaining their informed consent to participate in our study".

IRB: IRB approval - "This study was approved by the NHG Domain Specific Review Board in Singapore. NHG DSRB Ref: 2022/00479"; Informed Consent - ""The data collectors first approach potential participants to explain the study before obtaining their informed consent to participate in our study". Data protection is not discussed in this paper, but detailed in the published protocol on JMIR Research Protocols.

Development and pre-testing: The questionnaire was adapted from the World Health Organization's multi-country public awareness survey on antibiotic resistance, as described in the published protocol on JMIR Research Protocols.

Recruitment process and description of the sample having access to the questionnaire: Closed survey - Control Group: "A study team member (i.e., data collector) sends participants a detailed WhatsApp message six weeks after their recruitment date to inform them to complete the post-intervention KAP questionnaire", Intervention Group: "Six weeks after participants complete the intervention, a study team member sends a detailed WhatsApp message to inform them to complete the post-intervention KAP questionnaire."; Initial Contact - "Trained data collectors were employed to recruit participants in the Polyclinics. The data collectors first approach potential participants to explain the study before obtaining their informed consent to participate in our study." The surveys was not advertised to the public, only those who had given informed consent were provided with the survey(s).

Survey administration: E-survey sent out through WhatsApp - Control Group: "A study team member (i.e., data collector) sends participants a detailed WhatsApp message six weeks after their recruitment date to inform them to complete the post-intervention KAP questionnaire", Intervention Group: "Six weeks after participants complete the intervention, a study team member sends a detailed WhatsApp message to inform them to complete the post-intervention KAP questionnaire."; Incentives - "We reimbursed participants a small fee for completing the surveys"; Timeframe - Control Group: "six weeks after their recruitment date", Intervention Group: "Six weeks after participants complete the intervention"; Number of items - "Knowledge was measured via 11 True/False questions, while attitudes and perceptions were measured via 14 questions on a three-point Likert scale", "For the intervention group, satisfaction with the “SteWARdS Antibiotic Defence” app was measured with four questions on a five-point Likert scale and the reason(s) for recommending/not recommending the app to others and suggestions for improvement were obtained via open-ended questions, upon quest completion."

Response rates: Completion rate - "Of these randomly assigned participants, 206/240 (85.8%) in the control group and 142/240 (59.1%) in the intervention group completed the study and had their outcomes assessed"

Preventing multiple entries from the same individual: Each participant was assigned with a unique participant ID to track any duplicate entries

unique participant ID to track any duplicate entries.

Analysis: Handling of incomplete questionnaires - participants would not be able to submit incomplete questionnaires.

6a-ii) Describe whether and how "use" (including intensity of use/dosage) was defined/measured/monitored

Describe whether and how "use" (including intensity of use/dosage) was defined/measured/monitored (logins, logfile analysis, etc.). Use/adoption metrics are important process outcomes that should be reported in any ehealth trial.

	1	2	3	4	5	
subitem not at all important	○	○	◉	○	○	essential

Clear selection

Does your paper address subitem 6a-ii?

Copy and paste relevant sections from manuscript text

NA, user timing and interactions are not discussed in this paper as "our app usage data showed that participants who did not complete the game quest had too few interactions with the app to derive any benefit from the intervention."

6a-iii) Describe whether, how, and when qualitative feedback from participants was obtained

Describe whether, how, and when qualitative feedback from participants was obtained (e.g., through emails, feedback forms, interviews, focus groups).

	1	2	3	4	5	
subitem not at all important	○	○	○	○	◉	essential

Clear selection

Does your paper address subitem 6a-iii?

Copy and paste relevant sections from manuscript text

"For the intervention group, satisfaction with the “SteWARdS Antibiotic Defence” app was measured with four questions on a five-point Likert scale and the reason(s) for recommending/not recommending the app to others and suggestions for improvement were obtained via open-ended questions, upon quest completion."

6b) Any changes to trial outcomes after the trial commenced, with reasons

Does your paper address CONSORT subitem 6b? *

Copy and paste relevant sections from the manuscript (include quotes in quotation marks "like this" to indicate direct quotes from your manuscript), or elaborate on this item by providing additional information not in the ms, or briefly explain why the item is not applicable/relevant for your study

NA, primary and secondary outcomes remained the same throughout the trial, as we had intended to investigate.

7a) How sample size was determined

NPT: When applicable, details of whether and how the clustering by care provides or centers was addressed

7a-i) Describe whether and how expected attrition was taken into account when calculating the sample size

Describe whether and how expected attrition was taken into account when calculating the sample size.

	1	2	3	4	5	
subitem not at all important	○	○	○	○	◉	essential

Clear selection

Does your paper address subitem 7a-i?

Copy and paste relevant sections from manuscript title (include quotes in quotation marks "like this" to indicate direct quotes from your manuscript), or elaborate on this item by providing additional information not in the ms, or briefly explain why the item is not applicable/relevant for your study

"Assuming an effect size of 0.334 (95% CI: 0.260, 0.407) based on other serious games studies18, we estimated a minimum sample size of 142 per group to detect a significant change in the knowledge score at a power of 80% and an alpha level of 0.05. We initially planned to recruit 200 participants per arm to account for a 30% attrition rate. We later increased the recruitment numbers to 240 per arm to account for a 40% attrition rate as we observed higher-than-expected dropout rates."

7b) When applicable, explanation of any interim analyses and stopping guidelines

Does your paper address CONSORT subitem 7b? *

Copy and paste relevant sections from the manuscript (include quotes in quotation marks "like this" to indicate direct quotes from your manuscript), or elaborate on this item by providing additional information not in the ms, or briefly explain why the item is not applicable/relevant for your study

NA, no interim analyses were conducted and the intervention did not stop during the trial.

8a) Method used to generate the random allocation sequence

NPT: When applicable, how care providers were allocated to each trial group

Does your paper address CONSORT subitem 8a? *

Copy and paste relevant sections from the manuscript (include quotes in quotation marks "like this" to indicate direct quotes from your manuscript), or elaborate on this item by providing additional information not in the ms, or briefly explain why the item is not applicable/relevant for your study

Block randomization - "Eligible participants were 1:1 blocked randomized into the intervention or control group. Each block comprised two intervention and two control allocations. Participants determined their allocation by picking cards in the block, which was reset after all cards were drawn."

8b) Type of randomisation; details of any restriction (such as blocking and block size)

Does your paper address CONSORT subitem 8b? *

Copy and paste relevant sections from the manuscript (include quotes in quotation marks "like this" to indicate direct quotes from your manuscript), or elaborate on this item by providing additional information not in the ms, or briefly explain why the item is not applicable/relevant for your study

Block randomization - "Eligible participants were 1:1 blocked randomized into the intervention or control group. Each block comprised two intervention and two control allocations. Participants determined their allocation by picking cards in the block, which was reset after all cards were drawn."

9) Mechanism used to implement the random allocation sequence (such as sequentially numbered containers), describing any steps taken to conceal the sequence until interventions were assigned

Does your paper address CONSORT subitem 9? *

Copy and paste relevant sections from the manuscript (include quotes in quotation marks "like this" to indicate direct quotes from your manuscript), or elaborate on this item by providing additional information not in the ms, or briefly explain why the item is not applicable/relevant for your study

"Participants determined their allocation by picking cards in the block, which was reset after all cards were drawn"

10) Who generated the random allocation sequence, who enrolled participants, and who assigned participants to interventions

Does your paper address CONSORT subitem 10? *

Copy and paste relevant sections from the manuscript (include quotes in quotation marks "like this" to indicate direct quotes from your manuscript), or elaborate on this item by providing additional information not in the ms, or briefly explain why the item is not applicable/relevant for your study

"Participants determined their allocation by picking cards in the block, which was reset after all cards were drawn."

11a) If done, who was blinded after assignment to interventions (for example, participants, care providers, those assessing outcomes) and how

NPT: Whether or not administering co-interventions were blinded to group assignment

11a-i) Specify who was blinded, and who wasn't

Specify who was blinded, and who wasn't. Usually, in web-based trials it is not possible to blind the participants [1, 3] (this should be clearly acknowledged), but it may be possible to blind outcome assessors, those doing data analysis or those administering co-interventions (if any).

	1	2	3	4	5	
subitem not at all important	○	○	○	○	◉	essential

Clear selection

Does your paper address subitem 11a-i? *

Copy and paste relevant sections from the manuscript (include quotes in quotation marks "like this" to indicate direct quotes from your manuscript), or elaborate on this item by providing additional information not in the ms, or briefly explain why the item is not applicable/relevant for your study

"Participants were not blinded to the intervention as the data collectors had to explain to participants assigned to the intervention group about the "SteWARdS Antibiotic Defence" app. Apart from the data collectors, all other study team members (including the data analyst) were blinded to the group assignment."

11a-ii) Discuss e.g., whether participants knew which intervention was the "intervention of interest" and which one was the "comparator"

Informed consent procedures (4a-ii) can create biases and certain expectations - discuss e.g., whether participants knew which intervention was the "intervention of interest" and which one was the "comparator".

	1	2	3	4	5	
subitem not at all important	○	○	○	○	◉	essential

Clear selection

Does your paper address subitem 11a-ii?

Copy and paste relevant sections from the manuscript (include quotes in quotation marks "like this" to indicate direct quotes from your manuscript), or elaborate on this item by providing additional information not in the ms, or briefly explain why the item is not applicable/relevant for your study

"Participants were not blinded to the intervention as the data collectors had to explain to participants assigned to the intervention group about the “SteWARdS Antibiotic Defence” app."

11b) If relevant, description of the similarity of interventions

(this item is usually not relevant for ehealth trials as it refers to similarity of a placebo or sham intervention to a active medication/intervention)

Does your paper address CONSORT subitem 11b? *

Copy and paste relevant sections from the manuscript (include quotes in quotation marks "like this" to indicate direct quotes from your manuscript), or elaborate on this item by providing additional information not in the ms, or briefly explain why the item is not applicable/relevant for your study

NA, only one intervention was implemented. The comparator (i.e. the control group) does not undergo any intervention.

12a) Statistical methods used to compare groups for primary and secondary outcomes

NPT: When applicable, details of whether and how the clustering by care providers or centers was addressed

Does your paper address CONSORT subitem 12a? *

Copy and paste relevant sections from the manuscript (include quotes in quotation marks "like this" to indicate direct quotes from your manuscript), or elaborate on this item by providing additional information not in the ms, or briefly explain why the item is not applicable/relevant for your study

"Univariate analyses were used for baseline comparisons and weighted least square regressions (weighted on pre-test scores) were used to assess the association between the intervention and KAP scores at 6–10-week post-intervention"

12a-i) Imputation techniques to deal with attrition / missing values

Imputation techniques to deal with attrition / missing values: Not all participants will use the intervention/comparator as intended and attrition is typically high in ehealth trials. Specify how participants who did not use the application or dropped out from the trial were treated in the statistical analysis (a complete case analysis is strongly discouraged, and simple imputation techniques such as LOCF may also be problematic [4]).

	1	2	3	4	5	
subitem not at all important	○	○	◉	○	○	essential

Clear selection

Does your paper address subitem 12a-i? *

Copy and paste relevant sections from the manuscript (include quotes in quotation marks "like this" to indicate direct quotes from your manuscript), or elaborate on this item by providing additional information not in the ms, or briefly explain why the item is not applicable/relevant for your study

"We took the per-protocol approach in analyzing the study data, as our app usage data showed that participants who did not complete the game quest had too few interactions with the app to derive any benefit from the intervention"

12b) Methods for additional analyses, such as subgroup analyses and adjusted analyses

Does your paper address CONSORT subitem 12b? *

Copy and paste relevant sections from the manuscript (include quotes in quotation marks "like this" to indicate direct quotes from your manuscript), or elaborate on this item by providing additional information not in the ms, or briefly explain why the item is not applicable/relevant for your study

"Univariate analyses were used for baseline comparisons and weighted least square regressions (weighted on pre-test scores) were used to assess the association between the intervention and KAP scores at 6–10-week post-intervention"

X26) REB/IRB Approval and Ethical Considerations [recommended as subheading under "Methods"] (not a CONSORT item)

X26-i) Comment on ethics committee approval

	1	2	3	4	5	
subitem not at all important	○	○	○	○	◉	essential

Clear selection

Does your paper address subitem X26-i?

Copy and paste relevant sections from the manuscript (include quotes in quotation marks "like this" to indicate direct quotes from your manuscript), or elaborate on this item by providing additional information not in the ms, or briefly explain why the item is not applicable/relevant for your study

"This study was approved by the NHG Domain Specific Review Board in Singapore. NHG DSRB Ref: 2022/00479"

x26-ii) Outline informed consent procedures

Outline informed consent procedures e.g., if consent was obtained offline or online (how? Checkbox, etc.?), and what information was provided (see 4a-ii). See [6] for some items to be included in informed consent documents.

	1	2	3	4	5	
subitem not at all important	○	○	◉	○	○	essential

Clear selection

Does your paper address subitem X26-ii?

Copy and paste relevant sections from the manuscript (include quotes in quotation marks "like this" to indicate direct quotes from your manuscript), or elaborate on this item by providing additional information not in the ms, or briefly explain why the item is not applicable/relevant for your study

"Trained data collectors were employed to recruit participants in the Polyclinics. The data collectors first approach potential participants to explain the study before obtaining their informed consent to participate in our study"

X26-iii) Safety and security procedures

Safety and security procedures, incl. privacy considerations, and any steps taken to reduce the likelihood or detection of harm (e.g., education and training, availability of a hotline)

	1	2	3	4	5	
subitem not at all important	○	○	◉	○	○	essential

Clear selection

Does your paper address subitem X26-iii?

Copy and paste relevant sections from the manuscript (include quotes in quotation marks "like this" to indicate direct quotes from your manuscript), or elaborate on this item by providing additional information not in the ms, or briefly explain why the item is not applicable/relevant for your study

Privacy considerations are not discussed in this paper, but are detailed in the published protocol on JMIR Research Protocols - "An Evidence-Based Serious Game App for Public Education on Antibiotic Use and Antimicrobial Resistance: Protocol of a Randomized Controlled Trial".

Minimal harm is expected from using the serious-game app as the information provided are not out of the ordinary, and no personal information is inputted into the app or the survey at any point of time. The app does not collect personal details.

RESULTS

13a) For each group, the numbers of participants who were randomly assigned, received intended treatment, and were analysed for the primary outcome

NPT: The number of care providers or centers performing the intervention in each group and the number of patients treated by each care provider in each center

Does your paper address CONSORT subitem 13a? *

Copy and paste relevant sections from the manuscript (include quotes in quotation marks "like this" to indicate direct quotes from your manuscript), or elaborate on this item by providing additional information not in the ms, or briefly explain why the item is not applicable/relevant for your study

"We block randomized 480 (32.0%) eligible participants into two arms. Of these randomly assigned participants, 206/240 (85.8%) in the control group and 142/240 (59.1%) in the intervention group completed the study and had their outcomes assessed."

13b) For each group, losses and exclusions after randomisation, together with reasons

Does your paper address CONSORT subitem 13b? (NOTE: Preferably, this is shown in a CONSORT flow diagram) *

Copy and paste relevant sections from the manuscript (include quotes in quotation marks "like this" to indicate direct quotes from your manuscript), or elaborate on this item by providing additional information not in the ms, or briefly explain why the item is not applicable/relevant for your study

"We approached 1985 patients and visitors of polyclinics over three months, and 1144 (57.6%) consented to take part in our study. Of these consenting participants, 470 (41.1%) did not own an Android phone, 100 (8.7%) did not meet our age requirements, 80 (7.0%) were not proficient in the English language, 7 (0.6%) had difficulty downloading the app, and 7 (0.6%) had visual/cognitive impairment. We block randomized 480 (32.0%) eligible participants into two arms. Of these randomly assigned participants, 206/240 (85.8%) in the control group and 142/240 (59.1%) in the intervention group completed the study and had their outcomes assessed. Ninety-eight intervention group participants did not complete the game quest (16 withdrew; 82 dropped out) in the app "

13b-i) Attrition diagram

Strongly recommended: An attrition diagram (e.g., proportion of participants still logging in or using the intervention/comparator in each group plotted over time, similar to a survival curve) or other figures or tables demonstrating usage/dose/engagement.

	1	2	3	4	5	
subitem not at all important	○	○	○	○	◉	essential

Clear selection

Does your paper address subitem 13b-i?

Copy and paste relevant sections from the manuscript or cite the figure number if applicable (include quotes in quotation marks "like this" to indicate direct quotes from your manuscript), or elaborate on this item by providing additional information not in the ms, or briefly explain why the item is not applicable/relevant for your study

A CONSORT flow diagram is included in the manuscript, under the Results section. Please kindly refer to Figure 1.

14a) Dates defining the periods of recruitment and follow-up

Does your paper address CONSORT subitem 14a? *

Copy and paste relevant sections from the manuscript (include quotes in quotation marks "like this" to indicate direct quotes from your manuscript), or elaborate on this item by providing additional information not in the ms, or briefly explain why the item is not applicable/relevant for your study

"Recruitment occurred between January 2023 – March 2023, and follow-up was completed by early June 2023". A CONSORT flow diagram is included in the manuscript, under the Results section. Please kindly refer to Figure 1.

14a-i) Indicate if critical “secular events” fell into the study period

Indicate if critical “secular events” fell into the study period, e.g., significant changes in Internet resources available or “changes in computer hardware or Internet delivery resources”

	1	2	3	4	5	
subitem not at all important	○	○	●	○	○	essential

Clear selection

Does your paper address subitem 14a-i?

Copy and paste relevant sections from the manuscript (include quotes in quotation marks "like this" to indicate direct quotes from your manuscript), or elaborate on this item by providing additional information not in the ms, or briefly explain why the item is not applicable/relevant for your study

No secular events fell into the study period.

14b) Why the trial ended or was stopped (early)

Does your paper address CONSORT subitem 14b? *

Copy and paste relevant sections from the manuscript (include quotes in quotation marks "like this" to indicate direct quotes from your manuscript), or elaborate on this item by providing additional information not in the ms, or briefly explain why the item is not applicable/relevant for your study

NA, trial was not stopped early.

15) A table showing baseline demographic and clinical characteristics for each group

NPT: When applicable, a description of care providers (case volume, qualification, expertise, etc.) and centers (volume) in each group

Does your paper address CONSORT subitem 15? *

Copy and paste relevant sections from the manuscript (include quotes in quotation marks "like this" to indicate direct quotes from your manuscript), or elaborate on this item by providing additional information not in the ms, or briefly explain why the item is not applicable/relevant for your study

Baseline characteristics of participants are included in the manuscript - Kindly refer to Table 1 under the Results section.

15-i) Report demographics associated with digital divide issues

In ehealth trials it is particularly important to report demographics associated with digital divide issues, such as age, education, gender, social-economic status, computer/Internet/ehealth literacy of the participants, if known.

	1	2	3	4	5	
subitem not at all important	○	○	●	○	○	essential

Clear selection

Does your paper address subitem 15-i? *

Copy and paste relevant sections from the manuscript (include quotes in quotation marks "like this" to indicate direct quotes from your manuscript), or elaborate on this item by providing additional information not in the ms, or briefly explain why the item is not applicable/relevant for your study

"The randomization produced comparable groups as there were no significant between-group differences in participants' sociodemographic characteristics at baseline. Participants had a mean (SD) age of 36.9 (12.2) years. Our participant profile fits Singapore's population profile in terms of ethnicity (72.4% Chinese; 17.5% Malays; 6.9% Indians), education level (36.8% had tertiary education), and housing type (78.6% residing in conventional public housing flats). There were also no significant between-group differences in the mean (SD) baseline knowledge score 8.4/11 (1.6), but the attitude-perception score was significantly higher in controls (9.6 (2.6)) compared with intervention participants (8.8 (3.0)). In addition, 60.1% of participants had no exposure to advertisements on antibiotic use at baseline."

16) For each group, number of participants (denominator) included in each analysis and whether the analysis was by original assigned groups

16-i) Report multiple "denominators" and provide definitions

Report multiple "denominators" and provide definitions: Report N's (and effect sizes) "across a range of study participation [and use] thresholds" [1], e.g., N exposed, N consented, N used more than x times, N used more than y weeks, N participants "used" the intervention/comparator at specific pre-defined time points of interest (in absolute and relative numbers per group). Always clearly define "use" of the intervention.

	1	2	3	4	5	
subitem not at all important	○	○	◉	○	○	essential

Clear selection

Does your paper address subitem 16-i? *

Copy and paste relevant sections from the manuscript (include quotes in quotation marks "like this" to indicate direct quotes from your manuscript), or elaborate on this item by providing additional information not in the ms, or briefly explain why the item is not applicable/relevant for your study

"We block randomized 480 (32.0%) eligible participants into two arms. Of these randomly assigned participants, 206/240 (85.8%) in the control group and 142/240 (59.1%) in the intervention group completed the study and had their outcomes assessed. "

16-ii) Primary analysis should be intent-to-treat

Primary analysis should be intent-to-treat, secondary analyses could include comparing only "users", with the appropriate caveats that this is no longer a randomized sample (see 18-i).

	1	2	3	4	5	
subitem not at all important	○	○	○	○	○	essential

Does your paper address subitem 16-ii?

Copy and paste relevant sections from the manuscript (include quotes in quotation marks "like this" to indicate direct quotes from your manuscript), or elaborate on this item by providing additional information not in the ms, or briefly explain why the item is not applicable/relevant for your study

Intention-to-treat analysis was not feasible - "We took the per-protocol approach in analyzing the study data, as our app usage data showed that participants who did not complete the game quest had too few interactions with the app to derive any benefit from the intervention"

17a) For each primary and secondary outcome, results for each group, and the estimated effect size and its precision (such as 95% confidence interval)

Does your paper address CONSORT subitem 17a? *

Copy and paste relevant sections from the manuscript (include quotes in quotation marks "like this" to indicate direct quotes from your manuscript), or elaborate on this item by providing additional information not in the ms, or briefly explain why the item is not applicable/relevant for your study

Change in knowledge scores - "The mean standardized knowledge score improved from 77.6% at baseline to 82.4% post-game and 83.5% 6-10 weeks post-intervention among intervention group participants, while controls had a modest improvement of 75.4% from baseline to 78.1% at 6-10 weeks post-baseline (Multimedia Appendix Figure 1). Intervention group participants showed greater improvement in knowledge compared with controls [between-group differences (95% CI): 0.58 (0.28, 0.87)] after accounting for age, educational level, and exposure to advertisements on antibiotics and AMR (Table 2)."

Change in attitude-perception scores - "The mean standardized attitudes-perception score improved from 62.6% at baseline to 77.7% post-intervention among intervention group participants, while controls had a modest improvement of 68.9% from baseline to 71.9% post-intervention (Multimedia Appendix Figure S1). The proportion of intervention group participants with the “correct” attitudes and perceptions improved for all 14 statements post-intervention (Multimedia Appendix Figure S2). However, intervention participants still had misconceptions about antibiotic use after completing the game quest. The proportion of participants with correct responses for the statement “I need antibiotics to recover from serious symptoms from the common cold and flu” increased from 29.6% pre- to 46.5% post-intervention, and for “I need antibiotics if I continue to have flu symptoms after two weeks” increased from 22.5% pre- to 38.7% post-intervention. Intervention group participants showed better attitudes-perception improvement compared with controls [0.98 (0.44, 1.52)] after accounting for marital status and ethnicity (Table 3)."

App satisfaction - "The majority of participants (>95%) agreed that the “SteWARdS Antibiotic Defence” app improved their awareness on antibiotic use and AMR. More than 70% agreed that the app is easy to use and enjoyable, and 85.2% would recommend the app to others (Table 4). "

17a-i) Presentation of process outcomes such as metrics of use and intensity of use

In addition to primary/secondary (clinical) outcomes, the presentation of process outcomes such as metrics of use and intensity of use (dose, exposure) and their operational definitions is critical. This does not only refer to metrics of attrition (13-b) (often a binary variable), but also to more continuous exposure metrics such as “average session length”. These must be accompanied by a technical description how a metric like a “session” is defined (e.g., timeout after idle time) [1] (report under item 6a).

	1	2	3	4	5	
subitem not at all important	○	○	●	○	○	essential

Clear selection

Does your paper address subitem 17a-i?

Copy and paste relevant sections from the manuscript (include quotes in quotation marks "like this" to indicate direct quotes from your manuscript), or elaborate on this item by providing additional information not in the ms, or briefly explain why the item is not applicable/relevant for your study

NA, process outcomes metrics are not presented in this manuscript.

17b) For binary outcomes, presentation of both absolute and relative effect sizes is recommended

Does your paper address CONSORT subitem 17b? *

Copy and paste relevant sections from the manuscript (include quotes in quotation marks "like this" to indicate direct quotes from your manuscript), or elaborate on this item by providing additional information not in the ms, or briefly explain why the item is not applicable/relevant for your study

"The mean standardized knowledge score improved from 77.6% at baseline to 82.4% post-game and 83.5% 6-10 weeks post-intervention among intervention group participants, while controls had a modest improvement of 75.4% from baseline to 78.1% at 6-10 weeks post-baseline (Multimedia Appendix Figure 1). Intervention group participants showed greater improvement in knowledge compared with controls [between-group differences (95% CI): 0.58 (0.28, 0.87)] after accounting for age, educational level, and exposure to advertisements on antibiotics and AMR (Table 2)."

18) Results of any other analyses performed, including subgroup analyses and adjusted analyses, distinguishing pre-specified from exploratory

Does your paper address CONSORT subitem 18? *

Copy and paste relevant sections from the manuscript (include quotes in quotation marks "like this" to indicate direct quotes from your manuscript), or elaborate on this item by providing additional information not in the ms, or briefly explain why the item is not applicable/relevant for your study

"Univariate analyses were used for baseline comparisons and weighted least square regressions (weighted on pre-test scores) were used to assess the association between the intervention and KAP scores at 6–10-week post-intervention. All statistical assumptions were checked to ensure the accuracy of analyses"

18-i) Subgroup analysis of comparing only users

A subgroup analysis of comparing only users is not uncommon in ehealth trials, but if done, it must be stressed that this is a self-selected sample and no longer an unbiased sample from a randomized trial (see 16-iii).

	1	2	3	4	5	
subitem not at all important	○	○	◉	○	○	essential

Clear selection

Does your paper address subitem 18-i?

Copy and paste relevant sections from the manuscript (include quotes in quotation marks "like this" to indicate direct quotes from your manuscript), or elaborate on this item by providing additional information not in the ms, or briefly explain why the item is not applicable/relevant for your study

NA, no subgroup analysis was conducted.

19) All important harms or unintended effects in each group

(for specific guidance see CONSORT for harms)

Does your paper address CONSORT subitem 19? *

Copy and paste relevant sections from the manuscript (include quotes in quotation marks "like this" to indicate direct quotes from your manuscript), or elaborate on this item by providing additional information not in the ms, or briefly explain why the item is not applicable/relevant for your study

Minimal harm was anticipated as the information in-app were not out of the ordinary and no personal details were collected in-app or in the survey(s) at any point of time. It was made known to all participants at the point of informed consent that they can stop playing the game app or withdraw from the research study at any point of time. As for details on data protection, it is detailed in the published protocol on JMIR Research Protocols - "An Evidence-Based Serious Game App for Public Education on Antibiotic Use and Antimicrobial Resistance: Protocol of a Randomized Controlled Trial". Ethics approval was obtained from the NHG Domain Specific Review Board.

19-i) Include privacy breaches, technical problems

Include privacy breaches, technical problems. This does not only include physical "harm" to participants, but also incidents such as perceived or real privacy breaches [1], technical problems, and other unexpected/unintended incidents. "Unintended effects" also includes unintended positive effects [2].

	1	2	3	4	5	
subitem not at all important	○	○	●	○	○	essential

Clear selection

Does your paper address subitem 19-i?

Copy and paste relevant sections from the manuscript (include quotes in quotation marks "like this" to indicate direct quotes from your manuscript), or elaborate on this item by providing additional information not in the ms, or briefly explain why the item is not applicable/relevant for your study

No personal details were collected in-app or in the survey(s) at any point of time. For more details on data protection, it is detailed in the published protocol on JMIR Research Protocols - "An Evidence-Based Serious Game App for Public Education on Antibiotic Use and Antimicrobial Resistance: Protocol of a Randomized Controlled Trial". Ethics approval was obtained from the NHG Domain Specific Review Board.

19-ii) Include qualitative feedback from participants or observations from staff/researchers

Include qualitative feedback from participants or observations from staff/researchers, if available, on strengths and shortcomings of the application, especially if they point to unintended/unexpected effects or uses. This includes (if available) reasons for why people did or did not use the application as intended by the developers.

	1	2	3	4	5	
subitem not at all important	○	○	○	○	◉	essential

Clear selection

Does your paper address subitem 19-ii?

Copy and paste relevant sections from the manuscript (include quotes in quotation marks "like this" to indicate direct quotes from your manuscript), or elaborate on this item by providing additional information not in the ms, or briefly explain why the item is not applicable/relevant for your study

"For the intervention group, satisfaction with the “SteWARdS Antibiotic Defence” app was measured with four questions on a five-point Likert scale and the reason(s) for recommending/not recommending the app to others and suggestions for improvement were obtained via open-ended questions, upon quest completion. "

DISCUSSION

22) Interpretation consistent with results, balancing benefits and harms, and considering other relevant evidence

NPT: In addition, take into account the choice of the comparator, lack of or partial blinding, and unequal expertise of care providers or centers in each group

22-i) Restate study questions and summarize the answers suggested by the data, starting with primary outcomes and process outcomes (use)

Restate study questions and summarize the answers suggested by the data, starting with primary outcomes and process outcomes (use).

	1	2	3	4	5	
subitem not at all important	○	○	◉	○	○	essential

Clear selection

Does your paper address subitem 22-i? *

Copy and paste relevant sections from the manuscript (include quotes in quotation marks "like this" to indicate direct quotes from your manuscript), or elaborate on this item by providing additional information not in the ms, or briefly explain why the item is not applicable/relevant for your study

Restate study questions - "We evaluated the effectiveness of an evidence-based serious game mobile app in improving the KAP of appropriate antibiotic use and AMR among Singapore residents", "We also assessed the effectiveness of the app in short-term knowledge retention at 6-10 weeks post-intervention, which has often been overlooked."

Summarize data - "We observed a significant effect size of 0.58 (95% CI: 0.28, 0.87) in knowledge improvement, which concurs with other serious game studies on chronic disease management among young people and healthy lifestyle promotion", "We also observed a significant effect size of 0.98 (95% CI: 0.44, 1.52) in attitude-perception improvement with the “SteWARdS Antibiotic Defence” app.", "Our “SteWARdS Antibiotic Defence” app received an overall positive rating"

22-ii) Highlight unanswered new questions, suggest future research

Highlight unanswered new questions, suggest future research.

	1	2	3	4	5	
subitem not at all important	○	○	○	○	◉	essential

Clear selection

Does your paper address subitem 22-ii?

Copy and paste relevant sections from the manuscript (include quotes in quotation marks "like this" to indicate direct quotes from your manuscript), or elaborate on this item by providing additional information not in the ms, or briefly explain why the item is not applicable/relevant for your study

"Future research should assess the effectiveness of interventions that facilitate long-term knowledge retention and long-lasting behavioural change in antibiotic use and AMR"

20) Trial limitations, addressing sources of potential bias, imprecision, and, if relevant, multiplicity of analyses

20-i) Typical limitations in ehealth trials

Typical limitations in ehealth trials: Participants in ehealth trials are rarely blinded. Ehealth trials often look at a multiplicity of outcomes, increasing risk for a Type I error. Discuss biases due to non-use of the intervention/usability issues, biases through informed consent procedures, unexpected events.

	1	2	3	4	5	
subitem not at all important	○	○	○	○	◉	essential

Clear selection

Does your paper address subitem 20-i? *

Copy and paste relevant sections from the manuscript (include quotes in quotation marks "like this" to indicate direct quotes from your manuscript), or elaborate on this item by providing additional information not in the ms, or briefly explain why the item is not applicable/relevant for your study

"A few limitations exist in our study. First, measurement errors in KAP scores might occur if intervention group participants had requested their friends or family members to help with study completion. Nonetheless, controls could also have had help in completing the post-intervention survey. Therefore, any bias is likely towards the null, which attenuates the intervention's true effect. Second, there is a ceiling effect for participants with near-perfect baseline scores, which could attenuate the positive effects of the intervention. Lastly, we included only attendees at two government-funded primary care clinics (polyclinics). ", "In addition, we noticed several older adults rejecting study participation due to a lack of interest in completing the mobile app quest. Their lack of interest implies an additional effort to change their mindsets, and serious-game interventions may not be the best modality for educating older adults on public health issues. Reminders were also integral in driving the app completion rate. Hence, the actual uptake rate may be lower in the population. "

21) Generalisability (external validity, applicability) of the trial findings

NPT: External validity of the trial findings according to the intervention, comparators, patients, and care providers or centers involved in the trial

21-i) Generalizability to other populations

Generalizability to other populations: In particular, discuss generalizability to a general Internet population, outside of a RCT setting, and general patient population, including applicability of the study results for other organizations

	1	2	3	4	5	
subitem not at all important	○	○	○	○	◉	essential

Clear selection

Does your paper address subitem 21-i?

Copy and paste relevant sections from the manuscript (include quotes in quotation marks "like this" to indicate direct quotes from your manuscript), or elaborate on this item by providing additional information not in the ms, or briefly explain why the item is not applicable/relevant for your study

"Despite the limitations in recruitment, the sociodemographic distribution of our participants is representative of the Singaporean population. Hence, our findings are generalizable to the rest of the country."

21-ii) Discuss if there were elements in the RCT that would be different in a routine application setting

Discuss if there were elements in the RCT that would be different in a routine application setting (e.g., prompts/reminders, more human involvement, training sessions or other co-interventions) and what impact the omission of these elements could have on use, adoption, or outcomes if the intervention is applied outside of a RCT setting.

	1	2	3	4	5	
subitem not at all important	○	○	○	○	◉	essential

Clear selection

Does your paper address subitem 21-ii?

Copy and paste relevant sections from the manuscript (include quotes in quotation marks "like this" to indicate direct quotes from your manuscript), or elaborate on this item by providing additional information not in the ms, or briefly explain why the item is not applicable/relevant for your study

"we noticed several older adults rejecting study participation due to a lack of interest in completing the mobile app quest. Their lack of interest implies an additional effort to change their mindsets, and serious-game interventions may not be the best modality for educating older adults on public health issues. Reminders were also integral in driving the app completion rate. Hence, the actual uptake rate may be lower in the population. "

OTHER INFORMATION

23) Registration number and name of trial registry

Does your paper address CONSORT subitem 23? *

Copy and paste relevant sections from the manuscript (include quotes in quotation marks "like this" to indicate direct quotes from your manuscript), or elaborate on this item by providing additional information not in the ms, or briefly explain why the item is not applicable/relevant for your study

"ClinicalTrials.gov Identifier: NCT05445414"

24) Where the full trial protocol can be accessed, if available

Does your paper address CONSORT subitem 24? *

Cite a Multimedia Appendix, other reference, or copy and paste relevant sections from the manuscript (include quotes in quotation marks "like this" to indicate direct quotes from your manuscript), or elaborate on this item by providing additional information not in the ms, or briefly explain why the item is not applicable/relevant for your study

The full trial protocol can be found here:
Huang Z, Tang WE, Guo H, Natarajan K, Lee TH, Yeo TW, et al. An Evidence-Based Serious Game App for Public Education on Antibiotic Use and Antimicrobial Resistance: Protocol of a Randomized Controlled Trial. JMIR Res Protoc. 2023 2023/3/28;12:e45833. doi: 10.2196/45833.

25) Sources of funding and other support (such as supply of drugs), role of funders

Does your paper address CONSORT subitem 25? *

Copy and paste relevant sections from the manuscript (include quotes in quotation marks "like this" to indicate direct quotes from your manuscript), or elaborate on this item by providing additional information not in the ms, or briefly explain why the item is not applicable/relevant for your study

"This work is supported by the National Centre for Infectious Diseases [NCID Catalyst Grant (FY2022HZ)]. "

X27) Conflicts of Interest (not a CONSORT item)

X27-i) State the relation of the study team towards the system being evaluated

In addition to the usual declaration of interests (financial or otherwise), also state the relation of the study team towards the system being evaluated, i.e., state if the authors/evaluators are distinct from or identical with the developers/sponsors of the intervention.

	1	2	3	4	5	
subitem not at all important	○	○	○	○	◉	essential

Clear selection

Does your paper address subitem X27-i?

Copy and paste relevant sections from the manuscript (include quotes in quotation marks "like this" to indicate direct quotes from your manuscript), or elaborate on this item by providing additional information not in the ms, or briefly explain why the item is not applicable/relevant for your study

"All authors report no potential conflicts of interest. "

About the CONSORT EHEALTH checklist

As a result of using this checklist, did you make changes in your manuscript? *

- ○ yes, major changes
- ◉ yes, minor changes
- ○ no

What were the most important changes you made as a result of using this checklist?

Amended the study title slightly

How much time did you spend on going through the checklist INCLUDING making changes in your manuscript *

6 hours

As a result of using this checklist, do you think your manuscript has improved? *

- ○ yes
- ◉ no
- ○ Other:

Would you like to become involved in the CONSORT EHEALTH group?

This would involve for example becoming involved in participating in a workshop and writing an "Explanation and Elaboration" document

- ○ yes
- ◉ no
- ○ Other:

Clear selection

Any other comments or questions on CONSORT EHEALTH

Your answer

STOP - Save this form as PDF before you click submit

To generate a record that you filled in this form, we recommend to generate a PDF of this page (on a Mac, simply select "print" and then select "print as PDF") before you submit it.

When you submit your (revised) paper to JMIR, please upload the PDF as supplementary file.

Don't worry if some text in the textboxes is cut off, as we still have the complete information in our database. Thank you!

Final step: Click submit !

Click submit so we have your answers in our database!

Submit Clear form

Never submit passwords through Google Forms.

This content is neither created nor endorsed by Google. Report Abuse - Terms of Service - Privacy Policy

Google Forms
